# Chemokine signaling synchronizes angioblast proliferation and differentiation during pharyngeal arch artery vasculogenesis

**DOI:** 10.1242/dev.200754

**Published:** 2022-12-05

**Authors:** Jie Liu, Mingming Zhang, Haojian Dong, Jingwen Liu, Aihua Mao, Guozhu Ning, Yu Cao, Yiyue Zhang, Qiang Wang

**Affiliations:** ^1^ State Key Laboratory of Membrane Biology, Institute of Zoology, University of Chinese Academy of Sciences, Chinese Academy of Sciences, Beijing 100101, China; ^2^ Division of Cell, Developmental and Integrative Biology, School of Medicine, South China University of Technology, Guangzhou 510006, China; ^3^ Department of Cardiology, Guangdong Cardiovascular Institute, Guangdong Provincial People's Hospital, Guangdong Academy of Medical Sciences, Guangzhou 510080, China

**Keywords:** Pharyngeal arch artery, Cell proliferation, Angioblast differentiation, *cxcr4a*, *cxcl12b*, Zebrafish

## Abstract

Developmentally, the great vessels of the heart originate from the pharyngeal arch arteries (PAAs). During PAA vasculogenesis, PAA precursors undergo sequential cell fate decisions that are accompanied by proliferative expansion. However, how these two processes are synchronized remains poorly understood. Here, we find that the zebrafish chemokine receptor Cxcr4a is expressed in PAA precursors, and genetic ablation of either *cxcr4a* or the ligand gene *cxcl12b* causes PAA stenosis. Cxcr4a is required for the activation of the downstream PI3K/AKT cascade, which promotes not only PAA angioblast proliferation, but also differentiation. AKT has a well-known role in accelerating cell-cycle progression through the activation of cyclin-dependent kinases. Despite this, we demonstrate that AKT phosphorylates Etv2 and Scl, the key regulators of angioblast commitment, on conserved serine residues, thereby protecting them from ubiquitin-mediated proteasomal degradation. Altogether, our study reveals a central role for chemokine signaling in PAA vasculogenesis through orchestrating angioblast proliferation and differentiation.

## Teaser

**Summary:** Genetic ablation of either *cxcr4a* or the ligand gene *cxcl12b* causes pharyngeal arch artery (PAA) stenosis in zebrafish, revealing a central role for chemokine signaling in PAA vasculogenesis through orchestrating angioblast proliferation and differentiation.

## INTRODUCTION

Pharyngeal arch arteries (PAAs) are formed during vascular development of vertebrate embryos when endothelial cells migrate and extend into the pharyngeal arches to connect the heart with the dorsal aorta (Hiruma et al., 2002; Nagelberg et al., 2015; Rana et al., 2014). These paired, bilateral arteries arise by vasculogenesis from *nkx2.5^+^* precursors that reside in the second heart field within the anterior lateral plate mesoderm (ALPM) (Paffett-Lugassy et al., 2013; Wang et al., 2017). After this process occurs in mammals, PAAs either regress or extensively remodel into the carotid arteries and great vessels of the heart, including the aorta and pulmonary arteries (Congdon, 1922; Hiruma et al., 2002). Defects in the formation or remodeling of PAAs ultimately lead to either devastating forms of congenital cardiovascular malformation or several nonlethal diseases, such as dizziness, vertigo and/or tinnitus (Hoffman and Kaplan, 2002; Kodo and Yamagishi, 2011; Psillas et al., 2007). However, the etiology of these cardiovascular diseases is largely unknown. Therefore, a better understanding of the molecular mechanisms behind PAA development remains of great scientific and clinical significance.

Although the complex remodeling process observed in mammals does not occur during zebrafish PAA development, the initial formation and patterning of these arteries are highly conserved across vertebrate classes (Anderson et al., 2008). Moreover, the transparent nature of zebrafish embryos allows the visualization of dynamic cellular behaviors during PAA morphogenesis (Anderson et al., 2008; Kameda, 2009; Paffett-Lugassy et al., 2013). For example, previous studies have shown that during mid-somitogenesis, *nkx2.5^+^* progenitor cells are specified in the ALPM. A proportion of these *nkx2.5^+^* cells are ventricular precursors, as they migrate medially shortly afterwards and contribute to the heart. In contrast, the rest of *nkx2.5^+^* progenitors remain laterally located, condensing into several pharyngeal clusters through a craniocaudal sequence (Paffett-Lugassy et al., 2013). The *nkx2.5^+^* progenitor cluster located in pharyngeal arch 2 gives rise to head muscles and cardiac outflow (Paffett-Lugassy et al., 2017), whereas those within pharyngeal arches 3-6 are specified into PAA progenitors. This initiates expression of another lineage-specific gene – *npas4l –*that encodes a PAS domain-containing bHLH transcription factor (Mao et al., 2021). Subsequently, these PAA progenitors undergo angioblast transition, which is indicated by reduced *nkx2.5* expression and the emergence of *etv2* (*etsrp*) and *scl* (*tal1*) expression*.* They then fully differentiate into aortic arch angioblasts with *tie1* expression, and ultimately mature into endothelial cells that establish well-organized vascular structures (Paffett-Lugassy et al., 2013).

To obtain the appropriate size, morphology and function, normal organogenesis requires coordinated regulation of cell proliferation and differentiation during embryo development (Nakajima et al., 2011; Nakajo et al., 2011). In particular, PAA cell proliferation accompanies the sequential steps of cell fate decisions (Mao et al., 2019; Meng et al., 2017; Paffett-Lugassy et al., 2013). Studies from our group and other groups have indicated that impaired cell proliferation during PAA formation leads to vascular stenosis (Mao et al., 2019; Meng et al., 2017). In addition, the abnormal differentiation of either PAA progenitors or angioblasts results in vascular dysfunction (Abrial et al., 2017; Mao et al., 2021). Despite this, the molecular cues synchronously governing proliferative cellular expansion and functional differentiation during PAA development remain poorly understood.

Chemokines are a large family of small secreted proteins that act through their classical G protein-coupled receptors and play a primary role during the process of leukocyte trafficking (Raz and Mahabaleshwar, 2009; Siekmann et al., 2009). Among the chemokines discovered to date, the stromal cell-derived factor 1 (Sdf1/Cxcl12) and its receptor (Cxcr4) have been well characterized across a wide range of developmental processes, such as cell migration, muscle patterning and heart development (Raz and Mahabaleshwar, 2009). In particular, Cxcl12 and Cxcr4 signaling has been found to play crucial roles in the establishment of organ-specific vascular systems (Ara et al., 2005; Cavallero et al., 2015; Katsumoto and Kume, 2011; Tachibana et al., 1998; Takabatake et al., 2009). As a result of gene duplication, two Cxcl12 ligands (Cxcl12a and Cxcl12b) and two Cxcr4 receptors (Cxcr4a and Cxcr4b) are expressed in zebrafish embryos (Raz and Mahabaleshwar, 2009). Interestingly, instead of the Cxcl12a/Cxcr4b axis, Cxcl12b/Cxcr4a signaling has been implicated in the formation of the lateral dorsal aorta, arterial-venous connections and coronary vessels (Bussmann et al., 2011; Harrison et al., 2015; Siekmann et al., 2009). The expression of *cxcr4a* has been observed in various arteries, including those found in the pharyngeal arches (Bussmann et al., 2011; Fujita et al., 2011; Siekmann et al., 2009), suggesting a potential role for chemokine signaling in PAA development. In this study, we further find a crucial function of chemokine signaling in governing and coordinating angioblast proliferation and differentiation during PAA morphogenesis.

## RESULTS

### Loss of *cxcr4a* causes PAA stenosis

**Fig. 1.**
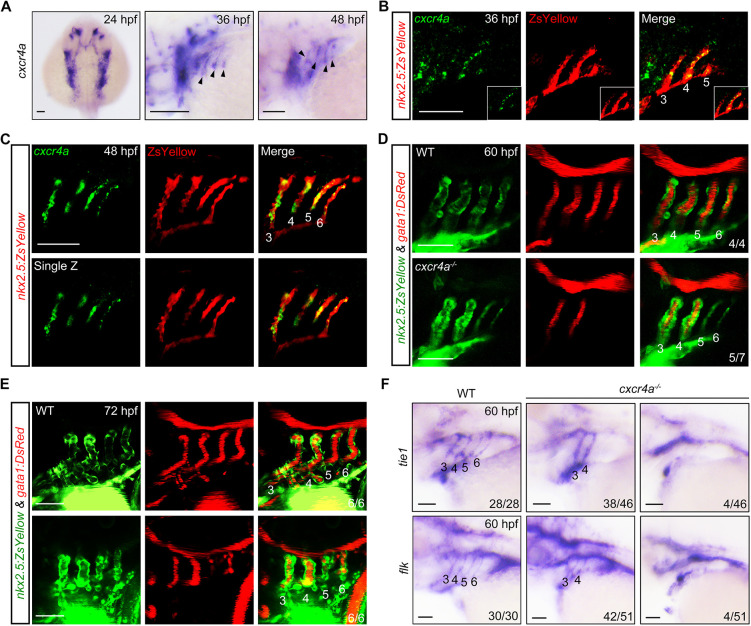
**Genetic depletion of *cxcr4a* induces PAA malformation.** (A) The expression of *cxcr4a* at 24 (dorsal view with anterior towards the top), 36 and 48 hpf (lateral views with anterior towards the left). Black arrowheads indicate the expression of *cxcr4a* in the pharynx. (B,C) Colocalization of *cxcr4a* transcripts and ZsYellow^+^ PAA angioblasts. *Tg(nkx2.5:ZsYellow)* embryos were stained with anti-ZsYellow antibody (red) and hybridized with *cxcr4a* probes (green) by fluorescent *in situ* hybridization. (D,E) Confocal images depicting blood flows in wild-type (WT) and *cxcr4a*-deficient embryos. PAAs 3-6 are marked with corresponding numbers. The ratios of affected embryos are indicated in the lower right corner. (F) The expression of *tie1* and *flk* in wild type (WT) and *cxcr4a^−/−^* mutants. Scale bars: 50 μm.

To explore the function of *cxcr4a* in PAA development, we examined its dynamic expression in the pharyngeal region. As early as 24 h post fertilization (hpf), high levels of *cxcr4a* transcripts were detected in the pharyngeal clusters (Fig. 1A). At later stages, *cxcr4a* showed a thin line-like expression pattern at sites corresponding to the developing PAAs (Fig. 1A). We further performed a detailed analysis using fluorescence *in situ* hybridization in *Tg(nkx2.5:ZsYellow)* embryos, where the developing aortic arches were labeled by fluorescence (Paffett-Lugassy et al., 2013). As shown in Fig. 1B,C, *cxcr4a* transcripts had a restricted distribution in PAA sprouts from 36 to 48 hpf, a developmental window that overlaps with the proliferation, differentiation and dorsal migration of PAA angioblasts (Mao et al., 2019).

We next analyzed the effects of loss of *cxcr4a* function on PAA formation in embryos carrying a targeted null mutation in this gene (Siekmann et al., 2009). Intriguingly, the majority of *cxcr4a* mutant embryos (5/7) had a much thinner morphology accompanied by a blockage of the blood flow in PAAs 5 and 6 at 60 hpf (Fig. 1D). A very small proportion of *cxcr4a^−/−^* embryos (1/7) displayed more-severe vascular stenosis, as indicated by the interruption of blood flow within in both the anterior and posterior PAAs. Importantly, when compared with control animals, *cxcr4a^−/−^* embryos still showed significantly less blood flow in PAAs 5-6 at 72 hpf (Fig. 1E), ruling out the possibility that the PAA defects were resulted from developmental delay. To further confirm the effect of *cxcr4a* on PAA development, we injected a previously validated morpholino (MO) that targets *cxcr4a* into embryos at the one-cell stage (Nair and Schilling, 2008). As expected, vascular stenosis appeared in the posterior PAAs of *cxcr4a* morphants (Fig. S1A). Consistent with these observations, the expression of two PAA endothelial cell markers – *tie1* and *flk* (*kdrl*) *–* was almost abolished in PAAs 5 and 6 of ∼80% *cxcr4a^−/−^* embryos at 60 hpf. Moreover, there was a significant decrease across all PAAs of ∼10% of mutants (Fig. 1F). Taken together, these results demonstrate a crucial role for *cxcr4a* in PAA vasculogenesis.

Our recently published studies uncovered an essential role for endodermal pouches in the development of adjacent pharyngeal tissues such as the brachial cartilages and PAAs (Li et al., 2019; Mao et al., 2021, 2019). However, *cxcr4a^−/−^* mutant embryos exhibited normally developed pouches and craniofacial cartilages (Fig. S1B,C), indicating that the *cxcr4a* deficient-induced PAA malformation may not be a secondary effect of impaired pouch and head skeleton development. Interestingly, it has been reported that *cxcr4a* is also expressed within the cranial neural crest cells (CNCCs), and *cxcr4a* morphants show aberrant defects in CNCC migration and craniofacial development (Olesnicky Killian et al., 2009). The discrepancy in the role of *cxcr4a* in head cartilage formation between our study and previous report may be due to genetic compensation in the *cxcr4a^−/−^* mutants (Rossi et al., 2015).

### Cxcr4a controls the proliferation and differentiation of PAA angioblasts

**Fig. 2.**
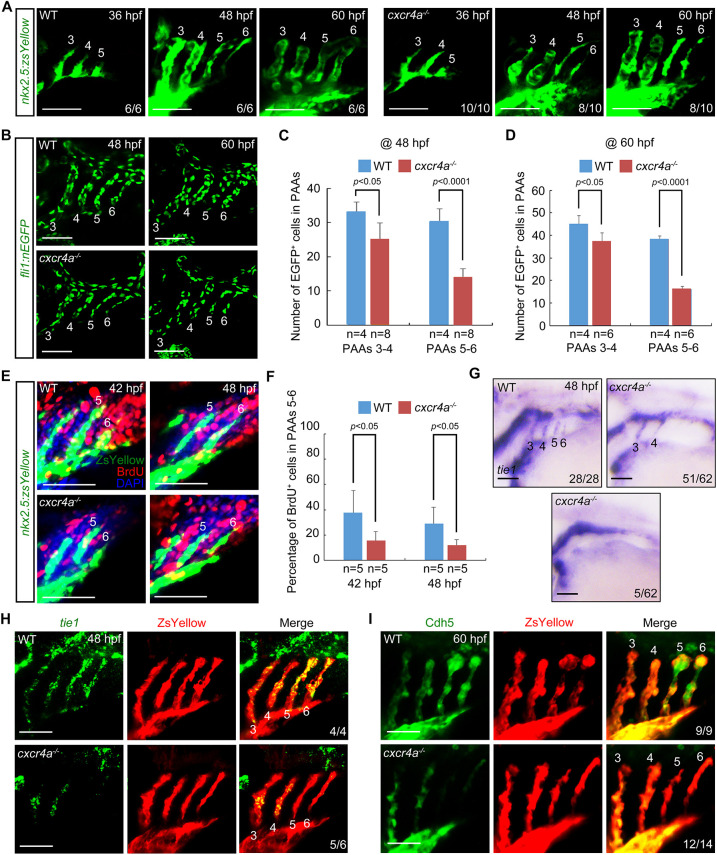
**Lack of *cxcr4a* represses both the proliferation and differentiation of PAA angioblasts.** (A) Time-lapse recording of ZsYellow fluorescence in the pharyngeal regions of wild-type (WT) and *cxcr4a^−/−^* embryos in *Tg(nkx2.5:ZsYellow)* background. (B-D) *cxcr4a^−/−^* mutants showed a significantly reduced cell number in posterior PAAs. Representative confocal sections of the PAAs in wild-type (WT) control and *cxcr4a-*deficient embryos are shown in B. Quantified cell numbers in PAAs 3-6 are shown in C and D. Error bars indicate s.d. of three biological replicates. (E,F) Detection of proliferative PAA angioblasts. Wild-type (WT) and *cxcr4a^−/−^* embryos were co-immunostained using anti-ZsYellow (green) and anti-BrdU (red) antibodies. Nuclear DNA was stained using DAPI (blue). Representative pictures are shown in E and the percentage of BrdU-positive cells in PAAs 5 and 6 for each group is shown in F. Error bars indicate s.d. of three biological replicates. (G,H) Expression analysis of *tie1* in the wild-type (WT) and *cxcr4a^−/−^* embryos at 48 hpf by *in situ* hybridization (G) and fluorescent *in situ* hybridization combined with immunostaining (H). (I) Cdh5 levels were greatly decreased in the posterior PAAs of *cxcr4a^−/−^* mutants. The indicated embryos were immunostained using Cdh5 (green) and ZsYellow (red) antibodies. Scale bars: 50 μm.

To dissect the cellular mechanisms underlying the PAA stenosis caused by *cxcr4a* deficiency, time-lapse image analysis was performed in *Tg(nkx2.5:ZsYellow)* embryos. In control animals, the PAA progenitors condensed into cluster 5 at 36 hpf, sprouted dorsally at 48 hpf, and finally organized into a vascular tube until 60 hpf (Fig. 2A). In *cxcr4a^−/−^* mutants, PAA cluster 5 was normally formed at 36 hpf; however, the corresponding angiogenic sprout appeared much thinner and failed to organize into a vascular lumen (Fig. 2A). Previous studies have shown that *cxcr4a* plays a role in guiding endothelial cell migration during the formation of lateral dorsal aorta (LDA), which carries blood flow that is essential for PAA development (Nicoli et al., 2010; Siekmann et al., 2009). Indeed, we found incomplete formation of the LDA in *cxcr4a^−/−^* mutants at 24 hpf (Fig. S2A). However, this defect was gradually restored before or at 48 hpf (Fig. S2A and B), and the blood flow within LDA appeared normal (Fig. 1D,E). Thus, the PAA abnormality in *cxcr4a^−/−^* embryos observed at 48 and 60 hpf may not be due to the earlier defects in LDA.

These above findings suggested the possibility that the arch artery stenosis in *cxcr4a^−/−^* mutants was related to a reduction in PAA cell number. To verify this, embryos expressing endothelial nuclear EGFP were used to assess *cxcr4a* effects on PAA cell numbers. Indeed, cell count numbers revealed a slight decrease in the two anterior PAAs along with a significant decline in the two posterior PAAs (Fig. 2B-D). Accordingly, loss of *cxcr4a* obviously reduced the diameters of PAAs 5 and 6 (Fig. S3). Likely reasons for the reduced PAA cell numbers in *cxcr4a^−/−^* embryos include delayed dorsal migration, increased apoptosis and/or decreased proliferation rate in the PAA precursors. We first examined PAA angioblast migration using a lineage-tracing analysis in *Tg(nkx2.5:Kaede)* embryos. The Kaede^+^ cells in PAA cluster 5 were photoconverted at 36 hpf. After conversion, their red derivatives were found throughout the PAA5 and sprouted into similar dorsal positions in both the wild-type and mutant embryos at 60 hpf (Fig. S4A,B). Furthermore, the PAA 5 and PAA 6 of *cxcr4a^−/−^* mutants fused and ultimately connected to the LDA at 72 hpf (Fig. S4C). These observations indicated that deletion of *cxcr4a* did not affect PAA cell migration. Meanwhile, TUNEL assays showed that loss of *cxcr4a* did not impair PAA angioblast survival (Fig. S4D). It has been shown that *cxcr4a* plays a major role in promoting endodermal cell proliferation (Stückemann et al., 2012). Therefore, we performed bromodeoxyuridine (BrdU) incorporation assays to examine whether *cxcr4a* also regulates the proliferation of PAA angioblasts. Coincidentally, we found a considerable decline in the proliferating ability of *cxcr4a*-depleted cells within PAAs 5 and 6 (Fig. 2E,F). Therefore, these results indicate that *cxcr4a* is required for angioblast proliferation, but dispensable for their migration and survival.

The failure of PAA lumen formation in the mutants raised the possibility that *cxcr4a* might also be crucial to cell fate commitment. To confirm this, we first analyzed the expression of the PAA progenitor marker gene *nkx2.5* by *in situ* hybridization at 38 hpf, and found no change upon *cxcr4a* depletion (Fig. S5A). We then evaluated the mRNA expression levels of *scl* and *etv2*, both of which are marker genes that indicate the early specification of the PAA angioblast lineage (Paffett-Lugassy et al., 2013). We observed that *cxcr4a* deficiency had no effect on the transition of PAA progenitors to angioblasts (Fig. S5B,C). Excitingly, we noted a dramatic reduction in expression of the PAA angioblast marker gene *tie1* in the posterior two sprouts in *cxcr4a^−/−^* mutants at 48 hpf (Fig. 2G,H), suggesting incomplete angioblast differentiation. Moreover, these poorly differentiated cells could not mature into functional endothelial cells, as revealed by the absence of Cdh5/VE-cadherin protein (Fig. 2I), a cell-cell adhesion molecule involved in endothelial cell polarity and vascular lumen formation (Charpentier and Conlon, 2014; Lampugnani et al., 2010; Montero-Balaguer et al., 2009). Therefore, *cxcr4a* plays key functions in promoting the proliferation and differentiation of PAA angioblasts.

### Cxcr4a activates downstream PI3K/AKT pathway to regulate angioblast proliferation and differentiation

**Fig. 3.**
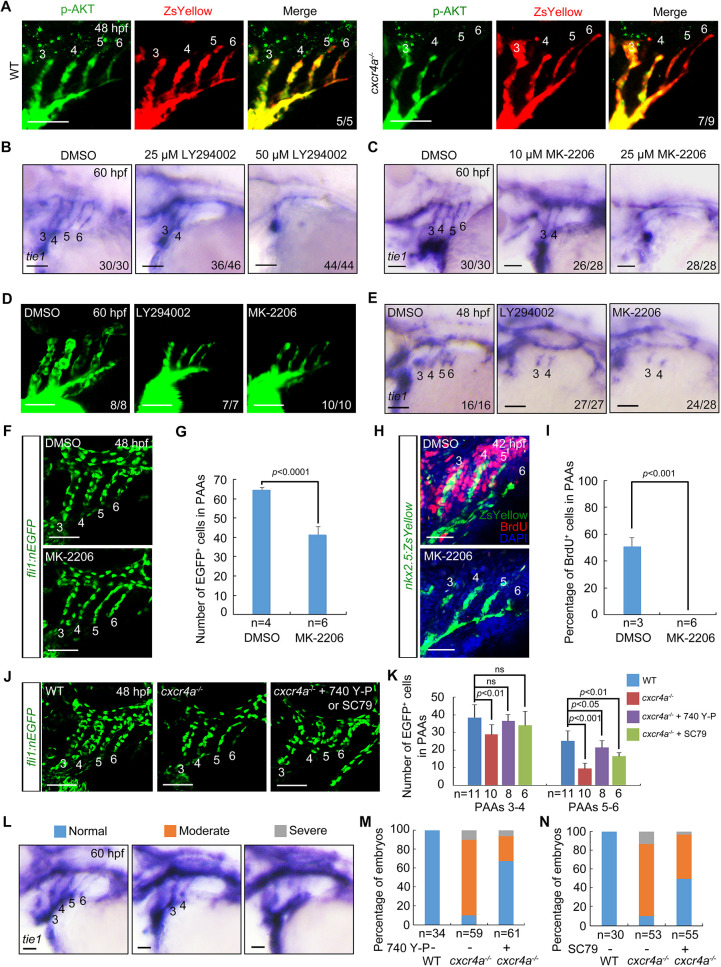
**Cxcr4a regulates PAA development through the PI3K/AKT cascade.** (A) AKT phosphorylation levels were decreased in PAAs 5 and 6 of *cxcr4a^−/−^* mutants. Control and *cxcr4a^−/−^*;*Tg(nkx2.5:ZsYellow)* embryos were immunostained using anti-p-AKT (green) and anti-ZsYellow (red) antibodies. (B,C) Wild-type embryos were treated with the PI3K inhibitor LY294002 (B) or the AKT inhibitor MK-2206 (C) from 18 hpf. Embryos were then harvested for *in situ* hybridization. (D) Live confocal images of *Tg(nkx2.5:ZsYellow)* embryos with 25 μM LY294002 or 10 μM MK-2206 from 18 to 60 hpf. (E) Embryos were treated with 25 μM LY294002 or 10 μM MK-2206 from 18 hpf until harvest for *in situ* hybridization. (F,G) Quantitative analysis of the number of PAA cells in embryos treated with either DMSO or 10 μM MK-2206. Representative pictures are shown in F and the number of PAA cells for each group is shown in G. Error bars indicate the s.d. of three biological replicates. (H,I) Confocal images of embryos treated with DMSO or 10 μM MK-2206 from 18 to 48 hpf. PAA angioblasts were labeled using antibody against ZsYellow (green), and proliferating cells were visualized by BrdU immunofluorescence (red) (H). Nuclei were stained with DAPI (blue). Ratios of BrdU-positive PAA cells were calculated and are shown in I. Error bars indicate the s.d. of three biological replicates. (J,K) Reduced cell number could be partially rescued by PI3K/AKT re-activation. *cxcr4a^−/−^* mutants were exposed to the PI3K agonist 740 Y-P (1 μM) or the AKT agonist SC79 (0.5 μM) from 18 to 48 hpf. Representative images are shown in J and quantified cell numbers are shown in K. Error bars indicate the s.d. of three biological replicates. Unpaired Student's *t*-test; ns, not significant. (L-N) *cxcr4a^−/−^* mutants were treated with 740 Y-P or SC79 from 18 to 60 hpf. Different phenotypes of PAAs were visualized using *tie1* expression (L). The percentages of affected embryos are shown in M,N. Scale bars: 50 μm.

CXCR4 exerts its biological effects by activating the downstream signaling pathways, IP3 [Ins(1,4,5)P_3_]/calcium, PI3K/AKT and ERK1/2 (Domanska et al., 2013; Duda et al., 2011). Although the IP3/calcium pathway functions primarily in cell survival, both PI3K/AKT and ERK1/2 signaling cascades contribute to cell proliferation and fate decisions (Domanska et al., 2013). To clarify which among the branch pathways correlated with Cxcr4a-regulated PAA development, *cxcr4a^−/−^* mutants were immunostained using antibodies against either phosphorylated AKT (p-AKT) or ERK1/2 (p-ERK1/2) at 48 hpf. We found no clear difference in the expression levels of p-ERK1/2 between wild-type and mutant animals (Fig. S6A). Conversely, p-AKT expression was profoundly repressed in the PAAs 3-6 of wild-type embryos treated with MK-2206, an allosteric AKT inhibitor (Hirai et al., 2010), and in the two posterior PAAs of *cxcr4a^−/−^* mutants (Fig. S6B; Fig. 3A). Furthermore, *cxcr4a* morphants also displayed similar decrease of p-AKT expression in the posterior PAAs as observed in *cxcr4a^−/−^* mutants (Fig. S6C). PI3K/AKT acts downstream of many signals, including Vegfα signaling that guides PAA angiogenesis (Nicoli et al., 2010). However, no significant difference in *vegfa* expression was found between wild-type embryos and *cxcr4a^−/−^* mutants (Fig. S6D). Thus, these results indicate a role for the PI3K/AKT pathway downstream of Cxcr4a during PAA morphogenesis.

LY294002 is a potent inhibitor of PI3K (Shan et al., 2008). Results from *in situ* hybridization experiments showed that wild-type embryos treated with either 25 μM LY294002 or 10 μM MK-2206 from 18 hpf exhibited a similar loss of *tie1* expression in PAAs 5 and 6 at 60 hpf relative to *cxcr4a*^−/−^ mutants (Fig. 3B,C). In particular, this effect was dose dependent, as higher concentrations led to almost completely abolished *tie1* expression in all PAAs (Fig. 3B,C), suggesting a different requirement for PI3K/AKT signal activity between the anterior and posterior PAAs. Notably, inhibitor-treated embryos also had PAA stenosis (Fig. 3D). Moreover, these pharmacological treatments did not disrupt the PAA progenitor-to-angioblast transition (Fig. S7A,B), but resulted in incomplete angioblast differentiation in PAA clusters 5 and 6 (Fig. 3E). To further confirm the direct effects of PI3K/AKT signal on the development of posterior PAAs, wild-type embryos were treated with LY294002 or MK-2206 from 36 hpf, when the progenitors in PAA clusters 3 and 4 have differentiated into angioblasts. As shown in Fig. S7C, the expression of *tie1* was almost abolished in PAAs 5 and 6 of inhibitor-treated embryos. Besides, treatment with these inhibitors led to reduced PAA cell number and diminished proliferative activity (Fig. 3F-I). Collectively, these mimicked the phenotypes observed in the *cxcr4a^−/−^* mutants.

Meanwhile, we treated the *cxcr4a^−/−^* mutants with either 740Y-P or SC79, which are potent agonists of PI3K and AKT, respectively (Hsieh et al., 2018; Wolman et al., 2015). Use of these agonists clearly restored the phosphorylation level of AKT, the numbers of PAA cells and the formation of PAAs in *cxcr4a^−/−^* mutants (Fig. S7D; Fig. 3J-N). Taken together, these results suggest that Cxcr4a promotes PAA development through its downstream PI3K/AKT signal cascade.

### AKT1 and its kinase activity are crucial to stabilize Etv2 and Scl

**Fig. 4.**
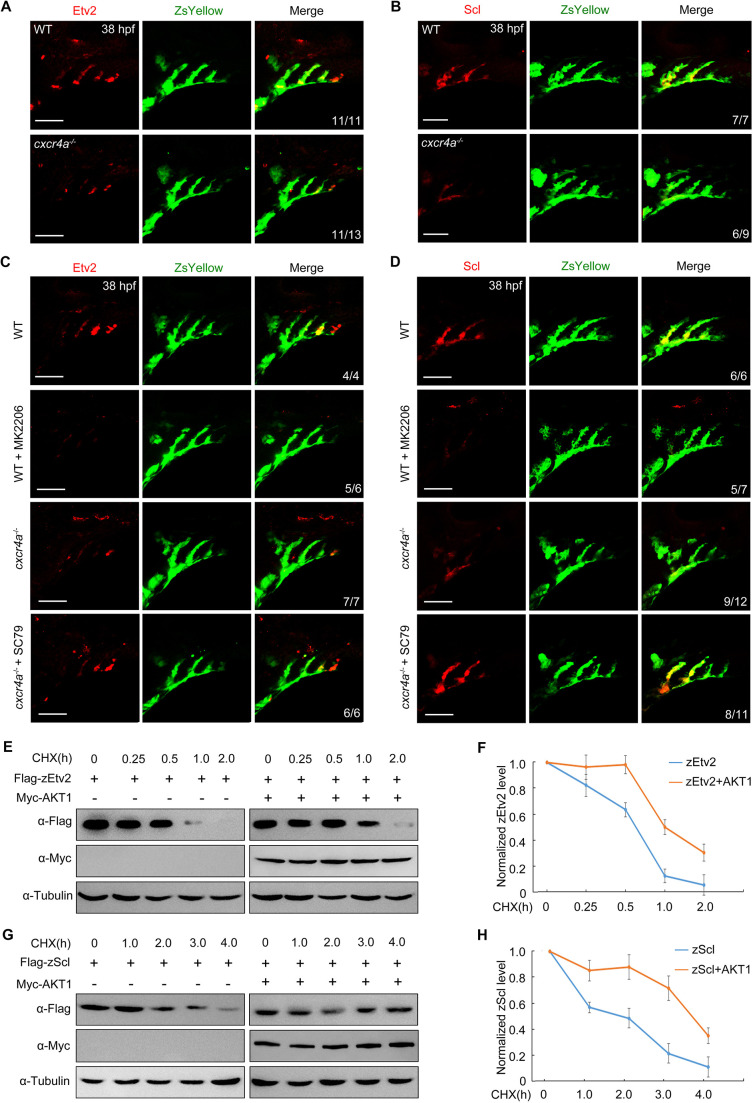
**AKT is required for stabilization of zEtv2 and zScl.** (A,B) Protein levels of zEtv2 and zScl were decreased in PAA clusters of *cxcr4a^−/−^* mutants. (C,D) Expression analysis of zEtv2 and zScl in wild type (WT) and *cxcr4a^−/−^* mutants on the *Tg(nkx2.5:ZsYellow)* background treated with 10 μM MK-2206 or 0.5 μM SC79 from 18 to 38 hpf. (E-H) HEK293T cells were transfected with indicated plasmids. (E,G) 24 h later, cells were treated with CHX (20 μg/ml) for the indicated times and harvested for immunoblotting. (F,H) Protein levels were quantified and normalized to tubulin (mean±s.d., three independent biological replicates). Scale bars: 50 μm.

Etv2 and Scl are evolutionarily conserved transcription factors that play key roles in angioblast commitment (Ren et al., 2010; Sumanas and Lin, 2006). As the mRNA levels of both *etv2* and *scl* were unchanged in mutant embryos, we assumed that their activity might be regulated at the post-translational level. To confirm this, we examined the protein expression levels of endogenous zebrafish Etv2 (zEtv2) and Scl (zScl). Immunofluorescence studies showed a significant decline in these protein levels in either all or posterior PAAs of *cxcr4a^−/−^* mutants (Fig. 4A,B). Moreover, treatment of wild-type embryos with the AKT-inhibitor MK-2206 yielded a significant decrease in zEtv2 and zScl expression (Fig. 4C,D). Notably, SC79-mediated reactivation of AKT in *cxcr4a^−/−^* embryos restored the expression of these proteins (Fig. 4C,D). Overall, these observations indicate that chemokine signaling has a function in regulating the protein stability of both zEtv2 and zScl through the PI3K/AKT pathway.

To further confirm the above conclusion, the effects of AKT expression on the protein degradation kinetics of zEtv2 and zScl were investigated in HEK293T cells. Results indicated that the co-expression of AKT1 significantly repressed the degradation rates of both zEtv2 and zScl proteins (Fig. 4E-H). In addition, activation of endogenous AKT in HeLa and HEK293T cells by SC79 notably increased zEtv2 and zScl expression levels (Fig. S8A-D). It is worth noting that the subcellular protein distribution of both zEtv2 and zScl remained unchanged after SC79 treatment (Fig. S8A and B). Thus, these findings indicate a crucial requirement of the Cxcr4a/PI3K/AKT signal cascade in promoting the stability of zEtv2 and zScl.

**Fig. 5.**
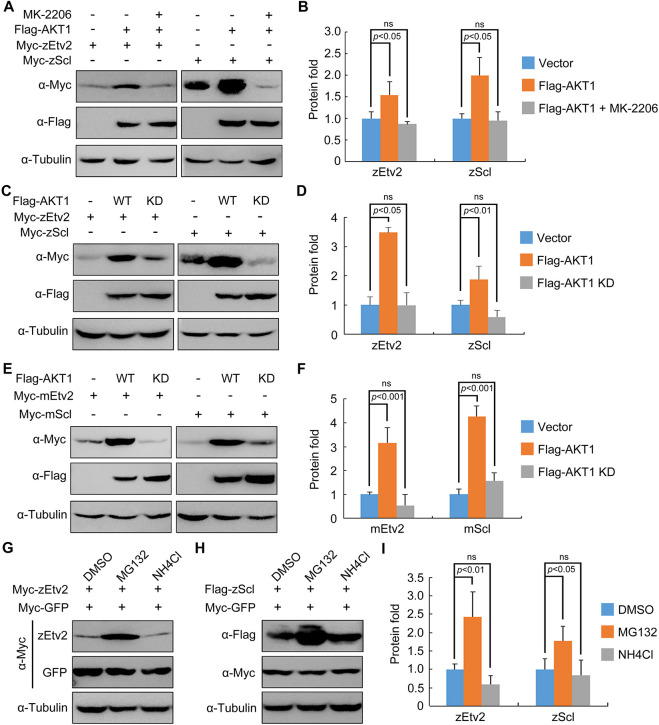
**AKT stabilizes zEtv2 and zScl through its kinase activity.** (A,B) HEK293T cells were co-transfected with plasmids expressing Flag-AKT1 and Myc-zEtv2 or -zScl, and treated with either DMSO or 0.5 μM MK-2206 for 24 h before undergoing western blot analysis. (C,D) Overexpression of wild-type (WT) AKT1 but not its kinase-deficient (KD) mutant stabilizes zEtv2 and zScl. (E,F) The promotion of mEtv2 or mScl expression level by AKT1 is dependent on its kinase activity. (G-I) zEtv2 and zScl proteins are degraded through the proteasome pathway. HEK293T cells co-transfected with plasmids encoding either Myc-zEtv2 or Flag-zScl and the negative control Myc-GFP were treated with either the lysozyme inhibitor NH_4_Cl (20 mM) or the proteasomal inhibitor MG132 (20 μM) for 5 h before harvest. In B,D,F,I, the gray values of the immunoreactive protein bands were quantified using ImageJ. Graphs show the density of the indicated protein signals relative to the corresponding Tubulin signals (mean±s.d., three independent biological repeats). Paired Student's *t*-test; ns, not significant.

We next asked whether the kinase activity of AKT is important to stabilizing zEtv2 and zScl proteins. To investigate this issue, HEK293T cells co-expressing Flag-AKT1 and Myc-zEtv2 were treated with the AKT kinase inhibitor MK-2206. Western blot analysis showed that the AKT1-induced increase of zEtv2 or zScl protein levels was abrogated by MK-2206 treatment (Fig. 5A,B). Consistently, the kinase-deficient form of AKT1, AKT1-T308A/S473A, lacked the ability to enhance both zEtv2 and zScl expression (Fig. 5C,D). Furthermore, wild-type AKT1 – but not its kinase-deficient mutant – was also able to promote mouse Etv2 and Scl expression (Fig. 5E,F). These data demonstrate that AKT1 and its kinase activity contribute to Etv2 and Scl protein stabilization across species.

In addition, as AKT plays a crucial role in the prevention of Etv2 and Scl protein degradation, MG132, a proteasome inhibitor – and NH4Cl – a lysosome inhibitor – were used to detect the pathway by which these proteins were degraded. Results indicated that treatment with MG132 resulted in dramatic stabilization of both zEtv2 and zScl (Fig. 5G-I). This suggests these two proteins are degraded through the proteasome pathway.

### AKT1 physically interacts with and directly phosphorylates Etv2 and Scl

Past studies have shown that the serine/threonine protein kinase AKT promotes cell-cycle progression through the G1 phase by phosphorylation and inactivation of cyclin-dependent kinase (CDK) inhibitors (Liang et al., 2002; Viglietto et al., 2002; Zhou et al., 2001). Given this, we hypothesized that lack of *cxcr4a* might lead to accumulation of angioblasts in the G1 phase. Therefore, we examined the effect of *cxcr4a* deficiency on cell-cycle progression in *Tg(EF1α:mKO2-zCdt1(1/190))* embryos, which expressed red nuclear fluorescence in cells in the G1 phase (Sugiyama et al., 2009). As expected, genetic inactivation of *cxcr4a* resulted in a markedly increased percentage of mKO2^+^ cells in PAAs (Fig. S9A,B), indicating that these cells were arrested in G1.

Progression through G1 and the G1/S transition are governed by CDK4/6 and CDK2 (Sherr and Roberts, 1999). Interestingly, these cell-cycle kinases are also characterized by their ability to control cell fate decisions by phosphorylating essential developmental regulators (Dumon et al., 2015; Liu et al., 2019; Nakajima et al., 2011; Pauklin and Vallier, 2013). With this in mind, wild-type *Tg(fli1:nucEGFP)* embryos were exposed to CYC202 and PD0332991, which are selective CDK2 and CDK4/6 inhibitors, respectively (De Azevedo et al., 1997; Fry et al., 2004). To our surprise and despite an apparent reduction in PAA cell numbers in the resulting embryos (Fig. S9C,D), the angioblast differentiation of PAA cells remained unaffected, as revealed by *tie1* expression (Fig. S9E). These observations demonstrate that both CDK2 and CDK4/6 are necessary for PAA cell proliferation, but are not required for angioblast differentiation. These results also raise the possibility that AKT might directly phosphorylate Etv2 and Scl to repress their turnover.

**Fig. 6.**
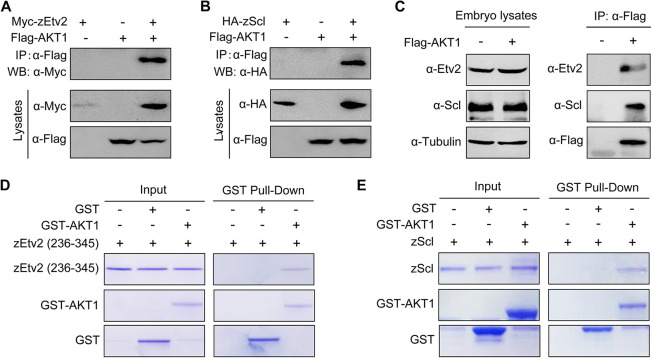
**AKT1 directly interacts with zEtv2 and zScl.** (A,B) AKT1 interacts with zEtv2 and zScl. HEK293T cells transfected with the indicated plasmids were harvested for immunoprecipitation with anti-Flag antibody and western blot analyses. (C) Overexpressed AKT1 interacts with endogenous zEtv2 and zScl. Wild-type embryos were injected with 200 pg *Flag-AKT1* mRNA at the one-cell stage, and then harvested for immunoprecipitation with anti-Flag antibody. (D,E) Direct binding of AKT1 to zEtv2 or zScl *in vitro*. GST, GST-AKT1, GST-zEtv2 (236-345) and GST-zScl were expressed in bacterial cells and purified. The purified GST-fusion proteins GST-zEtv2 (236-345) and GST-zScl were then treated with thrombin to cleave their GST tags, after which they were incubated with GST-AKT1. The pull-down fraction was separated with SDS-PAGE and analyzed by Coomassie Blue staining.

To explore whether Etv2 and Scl were substrates of AKT, we first performed co-immunoprecipitation experiments to detect possible interactions between these proteins. We found that Flag-tagged AKT1 associated with overexpressed or endogenous zEtv2 and zScl (Fig. 6A-C). This was further confirmed by the results of *in vitro* binding assays using recombinant proteins purified from *E. coli* (Fig. 6D,E). Therefore, these results indicate that AKT interacts directly with these two transcription factors.

**Fig. 7.**
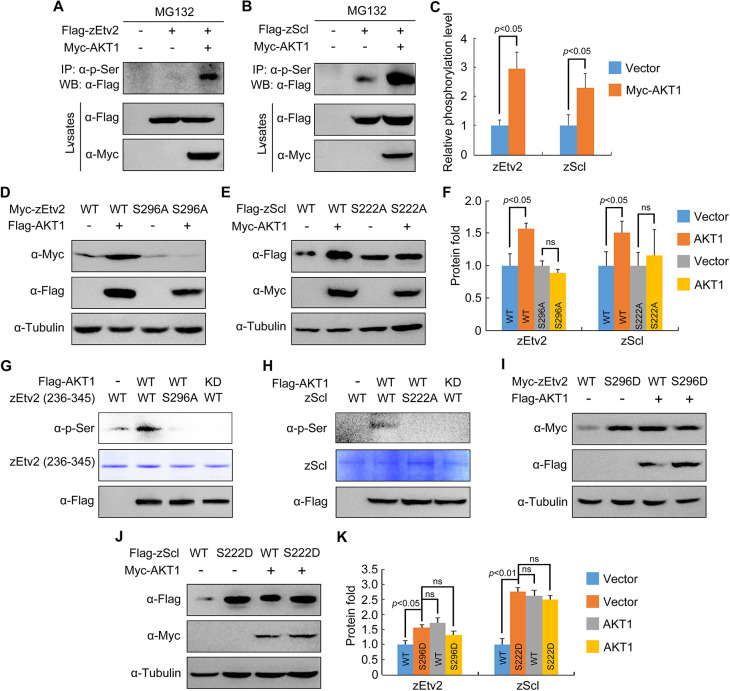
**AKT phosphorylates zEtv2 at S296 and zScl at S222.** (A-C) AKT increases the phospho-serine levels of zEtv2 and zScl. HEK293T cells were transfected with the indicated plasmids and treated with 20 μM MG132 for 5 h before harvest. Lysates were immunoprecipitated with anti-p-serine antibody and blotted with anti-Flag antibody. (D-F) The expression of wild-type zEtv2 and zScl – but not their phosphorylation-resistant mutants – was promoted by AKT1. (G,H) *In vitro* kinase assays revealed that AKT phosphorylated wild-type zEtv2 and zScl, but not their phosphorylation-resistant mutants. Flag-AKT1 was expressed in HEK293T cells and immunoprecipitated using anti-Flag-agarose beads. Wild-type and mutant proteins were purified from bacterial cells. The AKT-mediated phosphorylation of these purified proteins was detected by western blots using anti-p-serine antibody. (I-K) The phospho-mimicking mutants for zEtv2 and zScl exhibited greater stability than their wild-type proteins. HEK293T cells were transfected with the indicated plasmids and then harvested for immunoblotting. The zEtv2-S296D and zScl-S222D mutants were more stable than their corresponding wild-type forms, and were not further stabilized by AKT1. The gray values were quantified using ImageJ for quantification of phosphorylation levels (C) or protein expression levels (F,K) of zEtv2 and zScl (mean±s.d., three independent biological repeats). Paired Student's *t*-test; ns, not significant.

We then determined the role of AKT1 in the phosphorylation of zEtv2 and zScl. Myc-AKT1 was co-transfected into HEK293T cells with either Flag-tagged zEtv2 or zScl. Affinity-purified antibodies known to specifically recognize phosphoserine and phosphothreonine were used to enrich AKT substrates from whole-cell lysates. Western blot analysis showed that both the serine and threonine residues situated in zEtv2 and zScl were phosphorylated in the absence of overexpressed AKT1. However, only the phosphorylation of serine residues in zEtv2 and zScl was strongly induced by AKT1 overexpression (Fig. 7A-C; Fig. S10A,B). Thus, AKT interacts with and phosphorylates Etv2 and Scl on their respective serine residues.

It is widely known that AKT phosphorylates a variety of protein targets on serine and threonine residues within a consensus recognition motif of RXRXXS/T (Manning and Toker, 2017). However, we found that there was no such consensus substrate motif within zEtv2 and zScl. Considering the conserved effects of AKT on the protein stability of both Etv2 and Scl, we searched for potential phosphorylation sites on serine residues in both Etv2 and Scl by comparing homologous protein sequences from different species. As shown in Fig. S11A, zEtv2 shared two conserved serine residues (142 and 296).

To determine whether these identified serine sites were important for AKT-induced stabilization, we substituted them with alanine, creating S142A and S296A mutants. In the presence of AKT1, there was a notably increase in expression in the S142A mutant (Fig. S12A). Comparatively, this was nearly abolished in the S296A mutant (Fig. 7D,F). Similarly, ten conserved serine sites were identified within zScl and all of them were arranged into three groups (SI, SII and SIII) according to their respective position in the protein (Fig. S11B). Only when the serine residues (192 and 222) in the SII group were substituted with alanine did the zScl protein lose its response to AKT1 overexpression (Fig. S12B-D). We subsequently found that the expression of zScl carrying the S192A mutation was increased upon co-expression with AKT1 (Fig. S12E). However, the S222A mutant was not stabilized by AKT1 overexpression (Fig. 7E,F). Importantly, *in vitro* phosphorylation assays further showed that purified AKT1 phosphorylated zEtv2 on S296 and zScl on S222, respectively (Fig. 7G,H). Consistent with these observations, the phospho-mimicking mutants of zEtv2 (zEtv2-S296D) and zScl (zScl-S222D) were more stable than their wild-type proteins, and were not further stabilized by AKT1 overexpression (Fig. 7I-K). Taken together, these results indicate that AKT stabilizes Etv2 and Scl proteins through direct phosphorylation at specific serine residues.

### AKT stabilizes Etv2 and Scl by suppressing their polyubiquitylation

**Fig. 8.**
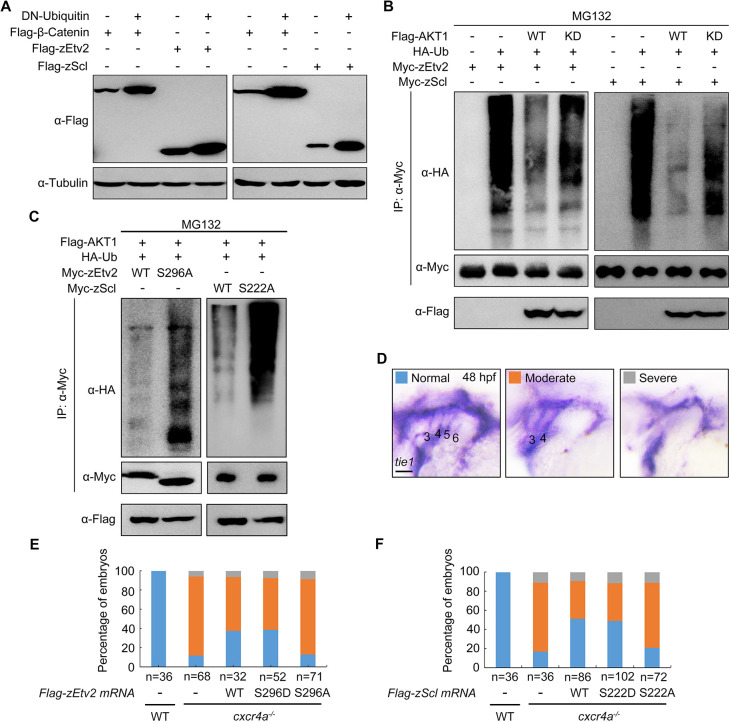
**AKT suppresses the polyubiquitylation of zEtv2 and zScl.** (A) zEtv2 and zScl degrade through the ubiquitin-proteasome pathway. Flag-tagged β-catenin, zEtv2 and zScl were co-expressed with Ub-K48R/G76A, a dominant-negative form of ubiquitin, in HEK293T cells. Lysates were then immunoblotted with the indicated antibodies. (B,C) AKT1 phosphorylates zEtv2 and zScl on specific serine residues to suppress their polyubiquitylation. HEK293T cells were transfected with the indicated constructs and incubated with 20 μM MG132 for 5 h before lysis. Ubiquitylated proteins were isolated by immunoprecipitation and ubiquitylation signals were detected by immunoblotting with an anti-HA antibody. Overexpression of wild-type (WT) AKT1, but not its kinase-deficient (KD) mutant, reduced the polyubiquitin modifications in zEtv2 and zScl proteins (B). Meanwhile, ectopic expression of AKT1 had a much more evident effect on the polyubiquitylation of the phosphorylation-resistant mutants of zEtv2 and zScl (zEtv2-S296A and zScl-S222A) compared with that of the relevant wild-type (WT) proteins (C). (D-F) Overexpression of either zEtv2/zScl-WT or their S-D form proteins in *cxcr4a^−/−^* mutants partially rescued *tie1* expression in PAAs 5 and 6. Wild-type (WT) or *cxcr4a^−/−^* mutant embryos were injected with 200 pg of *zEtv2/zScl-WT* or their S-D or S-A form mRNA and AS-Flag-photo-MO at the one-cell stage. After treatment with 365 nm UV for 10 min at 30 hpf, the injected mRNA was overexpressed in the embryos along with AS-Flag-photo-MO degradation, and embryos were collected at 48 hpf for *in situ* hybridization (D). Scale bars: 50 μm. The percentages of embryos with defects in *tie1* expression are shown in E,F.

As most protein substrates destined for degradation by the proteasome are tagged with covalently linked polyubiquitin chains (Muratani and Tansey, 2003), we wondered whether Etv2 and Scl could be modified with ubiquitin (Ub) molecules. By overexpressing Ub K48R/G76A, a dominant-negative inhibitor of poly-Ub chain formation (Finley et al., 1994; Ju and Xie, 2004), we found that the decay of zEtv2 and zScl proteins was distinctly repressed with an efficiency comparable with that of β-catenin (Fig. 8A), which is destined for ubiquitin-mediated degradation when Wnt signal is absent (Liu et al., 2002).

To further dissect whether AKT – together its kinase activity – regulates Etv2 and Scl polyubiquitylation, Flag-tagged wild-type AKT1 or its kinase-deficient mutant was transfected into HEK293T cells expressing HA-Ub and either Myc-tagged zEtv2 or zScl. Indeed, zEtv2 and zScl were both found to be heavily polyubiquitylated in the absence of Flag-AKT1 (Fig. 8B). Meanwhile, the polyubiquitin modifications in zEtv2 and zScl proteins were eliminated in the presence of overexpressed wild-type AKT1, but not its kinase-deficient mutant (Fig. 8B). This observation indicates that AKT inhibits the polyubiquitylation of zEtv2 and zScl by phosphorylation. Furthermore and upon ectopic expression of AKT1, the phosphorylation-resistant mutants of zEtv2 and zScl (zEtv2-S296A and zScl-S222A) were more efficiently polyubiquitylated when compared with the relevant wild-type proteins (Fig. 8C). These data strongly support the hypothesis that AKT1 phosphorylates Etv2 and Scl on their conserved serine residues – specifically, S296 in zEtv2 and S222 in zScl. Moreover, that this phosphorylation serves to suppress polyubiquitylation, thereby protecting them from ubiquitin-mediated proteasomal degradation.

Next, we sought to understand whether AKT1-induced stabilization of either zEtv2 or zScl was responsible for PAA angioblast differentiation. To do so, we used a previously described antisense photo-cleavable morpholino that targeted the N-terminal Flag sequence (AS-Flag-photo-MO) of *Flag-zEtv2* and *Flag-zScl* mRNAs to block their early translation (Wei et al., 2017). *cxcr4a^−/−^* mutants were then injected with a mixture containing AS-Flag-photo-MO and mRNA encoding either Flag-zEtv2 or its mutants. Afterwards, embryos were then exposed to UV at 30 hpf to relieve the blocking of mRNA translation. This temporal ectopic expression of either wild-type zEtv2 or its phospho-mimicking mutant (zEtv2-S296D) increased the percentage of *cxcr4a^−/−^* embryos with recovered *tie1* expression from 10% to 40% at 48 hpf (Fig. 8D,E). In contrast, overexpression of zEtv2-S296A – the phosphorylation-resistant mutant – had no palpable rescue effects (Fig. 8D,E). Likewise, the phosphorylation-resistant mutant (zScl-S222A) of zScl also lost its ability to restore *tie1* expression in *cxcr4a^−/−^* embryos (Fig. 8D,F). Together, these data suggest an important connection between AKT1-mediated stabilization of both Etv2 and Scl, and subsequent PAA angioblast differentiation.

### Cxcl12b is important for PAA development

**Fig. 9.**
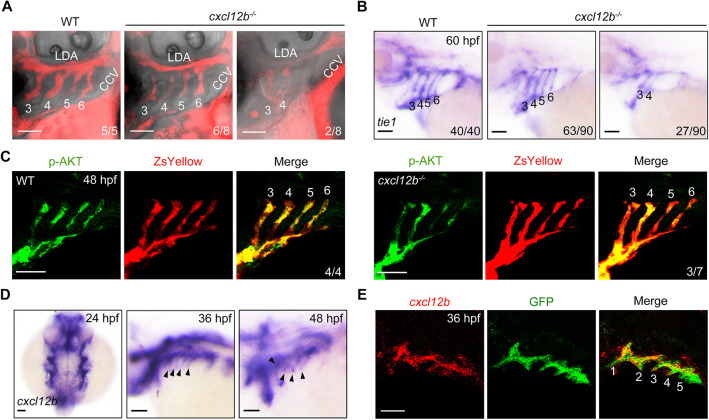
***cxcl12b* is expressed in pharyngeal pouches and is required for PAA morphogenesis.** (A) Deletion of *cxcl12b* led to an interruption of blood flow in the posterior PAAs. Wild-type (WT) embryos and *cxcl12b^−/−^* mutants were injected with rhodamine-dextran into the common cardinal vein at 72 hpf. The blood flow in PAAs was imaged using a Nikon A1R+ confocal microscope. (B) Analysis of *tie1* expression in wild-type (WT) embryos and *cxcl12b^−/−^* mutants. (C) Wild-type (WT) and *cxcl12b^−/−^* embryos on *Tg(nkx2.5:ZsYellow)* background were immunostained using anti-p-AKT (green) and anti-ZsYellow (red) antibodies. (D) Expression analysis of *cxcl12b* in wild-type embryos during PAA development. Black arrowheads indicate the expression of *cxcl12b* in the pharynx. (E) Colocalization analysis of *cxcl12b* transcripts with GFP proteins in pharyngeal pouches. *Tg(sox17:GFP)* transgenic embryos stained using anti-GFP (green) antibody were hybridized with *cxcl12b* probe (red). Scale bars: 50 μm.

We next asked whether Cxcl12b – the ligand of Cxcr4a in zebrafish – was involved in PAA formation. To directly examine regional blood flow in the pharynx, a fluorescent tracer (rhodamine-dextran, 2000 kDa) was injected into the common cardinal vein of both wild-type embryos and *cxcl12b* mutants. This mutant carried a frameshift mutation in the protein-coding sequence (Bussmann et al., 2011). As expected, *cxcl12b* inactivation resulted in dysplastic PAAs 5 and 6, as indicated by the interrupted blood flow (Fig. 9A). In parallel, the expression of *tie1* was lost in the posterior PAAs in *cxcl12b^−/−^* mutants at 60 hpf (Fig. 9B). However, *cxcl12b* depletion did not yield a fully penetrant phenotype, allowing relatively normal PAA development in ∼70% of embryos. Consistently, only some *cxcl12b^−/−^* mutants showed obviously reduced p-AKT level in the posterior PAAs (Fig. 9C). Combining these results with the previous finding that fewer than 50% of *cxcl12b* mutants showed defects in LDA formation (Bussmann et al., 2011), the observed lack of penetrance may be attributable to the activation of unknown compensatory mechanisms. Nevertheless, these results indicate that the Cxcl12b-Cxcr4a signaling pathway is crucial for PAA morphogenesis.

Interestingly, *cxcl12b* expression was present in the pharynx from 24 to 48 hpf (Fig. 9D). Furthermore, and as shown by the fluorescence *in situ* hybridization assay results in *Tg(sox17:GFP)* embryos, the expression of *cxcl12b* was highly restricted to the pouch endoderm adjacent to the developing PAAs (Fig. 9E). These results imply that pouch endoderm-expressed *cxcl12b* might be responsible for activation of Cxcr4a signaling in PAA angioblasts.

## DISCUSSION

Interconnections between proliferation and differentiation processes during organogenesis are tightly orchestrated by extrinsic cues from the niche microenvironments and intrinsic regulators that function to regulate the expression of genes important for cell cycle progression and cell fate determination (Dumon et al., 2015; Sincennes et al., 2016; Su et al., 2018; Widberg et al., 2009). PAA precursors sequentially differentiate into angioblasts as they rapidly proliferate, and both hypoproliferation and impaired differentiation lead to unsuccessful PAA morphogenesis (Abrial et al., 2017; Mao et al., 2019; Meng et al., 2017). We have shown here that PAA cell proliferation and differentiation are coupled at the molecular level. Cxcl12b-Cxcr4a signaling activates its downstream PI3K/AKT cascade to orchestrate PAA angioblast proliferation and differentiation. It has been reported that blood flow-triggered PI3K/AKT signaling is required for the correct migration of PAA cells (Nicoli et al., 2010). However, PAA angioblasts in *cxcr4a*^−/−^ mutants display no migration defects. PAAs 5 and 6 are lumenized by 50 hpf and exhibit blood flow by 52 hpf (Anderson et al., 2008; Nicoli et al., 2010). In our study, the p-AKT expression is profoundly reduced in the PAAs of *cxcr4a*^−/−^ mutants before or at 48 hpf. Thus, PI3K/AKT pathway may be activated by diversified upstream signals at different developmental stages and perform distinct functions during PAA morphogenesis.

AKT has been proposed to promote cell-cycle progression through the G1 phase by activation of CDKs and inactivation of CDK inhibitor p21^kip1^ and p27^kip1^ via phosphorylation (Liang et al., 2002; Viglietto et al., 2002; Zhou et al., 2001). Indeed, we found that *cxcr4a* deficiency resulted in a marked increase of PAA cells arrested in G1. We further demonstrated that AKT phosphorylates and stabilizes Etv2 and Scl by reducing their polyubiquitylation and subsequently promoting PAA angioblast differentiation. Coincidentally, and in support of our findings, AKT was found to synchronize cell proliferation and differentiation during erythropoiesis by phosphorylating GATA1, thereby increasing its affinity for FOG1 (Kadri et al., 2015). The AKT family of kinases includes three isoforms: AKT1, AKT2 and AKT3 (Manning and Toker, 2017). These AKT isoforms are functionally distinct, owing to differences in target specificity (Lee et al., 2014; Sanidas et al., 2014). Although AKT1 appears to be the major isoform that contributes to normal endothelial cell physiological functions, such as cell proliferation, migration and survival (Chen et al., 2005; Lee et al., 2014), further studies are needed to clarify which of these isoforms acts downstream of Cxcl12b-Cxcr4a signaling to regulate Etv2 and Scl stabilization during PAA formation.

AKT generally phosphorylates its substrates on serine and threonine residues within the minimal consensus recognition motif of RXRXXS/T (Lee et al., 2014; Manning and Toker, 2017; Sanidas et al., 2014). However, there was no such consensus AKT phosphorylation motif situated in either of the zEtv2 or zScl. Even so, systematic alanine mutagenesis showed that serine 296 of zEtv2 and serine 222 of zScl were the primary AKT phosphorylation sites. AKT-mediated phosphorylation of Etv2 and zScl at these conserved serine residues prevents polyubiquitin modifications in both zEtv2 and zScl, and protects them from ubiquitin-mediated proteasomal degradation. These phosphorylation events have potential functional consequences in PAA formation, as the phosphorylation-resistant mutants of zEtv2 and zScl lost their ability to rescue the defects of angioblast differentiation. A previous study has indicated that, in the human T-cell leukemia cell line Jurkat, transforming growth factor β (TGFβ) signaling triggers Scl polyubiquitylation and degradation through AKT-mediated phosphorylation at threonine 90 (Terme et al., 2009). Threonine 90 of human SCL lies within a consensus AKT phosphorylation motif, which is not present in the homologous zebrafish protein. Consistent with this, AKT did not enhance the phosphothreonine level of zScl. Collectively, these findings implied that the protein stability of Etv2 and SCL might be differentially regulated by AKT in distinct biological contexts.

PAA progenitors undergo a sequential series of cell fate decisions to generate functional arteries. Recent researches have highlighted the importance of cellular signaling pathways leading to PAA morphogenesis. For example, Gdf3/Alk4 signaling mediates the Tbx1-dependent emergence of *nkx2.5^+^* pharyngeal multipotent cells within the ALPM (Guner-Ataman et al., 2018). Moreover, BMP signaling is essential for the further specification of PAA progenitors from the *nkx2.5^+^* pharyngeal mesoderm (Mao et al., 2021), and TGF-β/Smad3 signaling is responsible for promoting PAA progenitor cell differentiation toward the angioblast lineage (Abrial et al., 2017). It is worth noting that our current study shows an indispensable role for Cxcl12b-Cxcr4a signaling in the later differentiation process after PAA angioblast transition. Genetic ablation of *cxcr4a* affected only the angioblast differentiation in PAAs 5 and 6 in most embryos, implying different sensitivities to the inactivation of Cxcr4a signaling between anterior and posterior PAAs. Interestingly, wild-type embryos treated with lower concentrations of either PI3K or AKT inhibitors led to a phenotype similar to *cxcr4a^−/−^* mutants. However, treatment with higher inhibitor concentrations induced significant defects in angioblast differentiation in all PAAs. These findings raise the possibility that, in addition to Cxcr4a signaling, there remain other, unidentified signaling mechanisms that activate the PI3K/AKT cascade and facilitate PAA formation.

Endodermal pouches are a series of outpocketings adjacent to the developing PAAs. These pouches have been found to express various signal molecules that guide PAA development. For example, previous studies from our lab have established that secreted BMP and PDGF ligands from pouches are required for PAA progenitor specification and angioblast proliferation (Mao et al., 2021; Mao et al., 2019). It has been reported that TGFβ2a and TGFβ3 are expressed at high levels in endodermal pouches; moreover, TGFβ signaling is necessary and sufficient for PAA angioblast differentiation (Abrial et al., 2017). *cxcl12b* is expressed in the pouch endoderm during PAA development, and its receptor gene *cxcr4a* is expressed in neighboring developing aortic arches. Interestingly, it seems that not all PAA angioblasts express *cxcr4a*, which may be because these cells are in different cell cycle phases or at different differentiation stages. Nonetheless, inactivation of *cxcl12b* leads to PAA defects similar to those observed in *cxcr4a^−/−^* mutants. These observations indicate a conceivable requirement for chemokine ligands from pharyngeal pouches in signal activation and PAA morphogenesis.

## MATERIALS AND METHODS

### Ethics statement

Our zebrafish experiments were all approved and carried out in accordance with the Animal Care Committee at the Institute of Zoology, Chinese Academy of Sciences (permission number: IOZ-13048).

### Zebrafish husbandry and strains

Zebrafish embryos were raised at 28.5°C in Holtfreter's solution and staged based on their morphology. For anesthesia or euthanasia, zebrafish embryos were immersed in fish water containing 0.4% tricaine (ethyl 3-aminobenzoate, E10505, Sigma).

We used the following mutant and transgenic lines: *Tg(nkx2.5:ZsYellow)* (Paffett-Lugassy et al., 2013), *Tg(gata1:DsRed)*, *Tg(fli1:nucEGFP)* (Meng et al., 2017), *Tg(nkx2.5:Kaede)* (Paffett-Lugassy et al., 2013), *Tg(EF1α:mKO2-zCdt1(1/190))* (Sugiyama et al., 2009), *Tg(flk:EGFP)*, *Tg(flk:mCherry)* and *Tg(sox17:GFP)*. Homozygous *cxcr4a^−/−^* and *cxcl12b^−/−^* mutants were identified from the offspring of heterozygous *cxcr4a^um20^* and *cxcl12b^mu100^* parents, respectively, through genotyping as previously described (Bussmann et al., 2011; Siekmann et al., 2009).

### Whole-mount *in situ* hybridization

Digoxigenin-UTP-labeled antisense RNA probes were transcribed using MEGAscript Kit (Ambion) according to the manufacturer's instructions. Whole-mount *in situ* hybridizations were performed according to previously published methods (Ning et al., 2013). In particular, fluorescent *in situ* hybridization in immunostained embryos was conducted as previously described (Mao et al., 2021). Briefly, for the detection of *cxcr4a* and *tie1* expression, *Tg(nkx2.5:ZsYellow)* embryos were first immunostained using affinity-purified anti-ZsYellow antibody (1:800; TA180004, Origene), and then subjected to *in situ* hybridization with digoxigenin-labeled *cxcr4a* or *tie1* probe. Anti-digoxigenin-HRP (1:400; Roche, 11633716001) was used as primary antibody to detect the probes and the hybridization signals were visualized by incubating embryos with fluorescein tyramide (1:50; PerkinElmer, NEL701A001KT). For the detection of *cxcl12b* expression, *Tg(sox17:GFP)* embryos were fluorescently stained with anti-GFP (1:1000; A11120, Invitrogen) antibody. The resulting hybridization signals were similarly generated with cyanine 3 tyramide (1:50; PerkinElmer, NEL701A001KT).

### Microinjection

Capped mRNAs for zEtv2, zScl and their mutant forms were synthesized *in vitro* from linearized plasmids using the mMessage mMachine kit (Ambion). The standard cMO (5′-CCTCTTACCTCAGTTACAATTTATA-3′), splicing MO targeting *cxcr4a* (5′-AGACGATGTGTTCGTAATAAGCCAT-3′) and AS-Flag-photo-MO (5′-TCATCGTCGTpCTTGTAGTCCAT-3′) were synthesized by Gene Tools and resuspended in nuclease-free water.

For the rescue experiments, *cxcr4a^−/−^* mutant embryos were co-injected with pre-mixed AS-Flag-photo-MO and either *Flag-zEtv2* or *Flag-zScl* mRNA (200 pg mRNA and 1 ng AS-Flag-photo-MO per embryo) at the one-cell stage. Embryos were then treated at 30 hpf with UV at 365 nm for 10 min using Lightbox (Gene Tools). All these experiments were conducted under constant dark conditions.

For the detection of blood flow, rhodamine-dextran (D7139, Invitrogen) was injected into the common cardinal vein of *cxcl12b^−/−^* mutants 10 min before 72 hpf. The embryos were then embedded with 2% low melting agarose and imaged using a Nikon A1R+ confocal microscope.

### Immunofluorescence staining

Embryos were fixed in 4% paraformaldehyde overnight, then rinsed with PBST four times every 5 min. Next, the resulting embryos were blocked at room temperature for at least 1 h in PBST containing 10% heat-inactivated goat serum and 1% BSA, and then stained using the following affinity-purified primary antibodies overnight at 4°C: anti-ZsYellow (1:200; 632475, Clontech), anti-ZsYellow (1:800; TA180004, Origene), anti-Cdh5 (1:200; 555289, BD Pharmingen), anti-pERK1/2 (1:1000; 9101, Cell Signaling), anti-pAKT (1:400; 4060, Cell Signaling Technology), anti-Scl (1:200; NBP2-50285, Novus Biologicals), anti-Etv2 (1:500; ES1004, Kerafast), anti-GFP (1:1000; A11120, Invitrogen) and anti-Flag (1:500; M20008, Abmart). Samples were then washed for 3-4 h with PBST, followed by incubation with secondary antibodies for 1 h at room temperature, including DyLight 488-conjugated goat anti-rabbit IgG (1:200; 711-545-152, Jackson), DyLight 594-conjugated goat anti-mouse IgG (1:200; 715-585-150, Jackson), DyLight 488-conjugated AffiniPure goat anti-mouse IgG (1:200; 715-545-150, Jackson) and DyLight 594-conjugated AffiniPure goat anti-rabbit IgG (1:200; 711-585-152, Jackson). DAPI (1:10,000, Sigma) was used as a nuclear stain.

### Pharmacological treatment

To block PI3K/AKT activity, embryos were treated with LY294002 (M1925, Abmole) or MK-2206 (sc-364537, Santa Cruz) at indicated concentrations from 18 or 36 hpf to the desired stages. For AKT inhibition in cultured cells, HEK293T cells were treated with 0.5 μM MK-2206 for 24 h before harvest. To activate PI3K/AKT activity, embryos were treated with 1 μM 740 Y-P (1236188-16-1, R&D Systems) or 0.5 μM SC79 (SF2730, Beyotime) from 18 hpf until harvest, respectively, and HeLa cells were treated with 10 μM SC79 for 2 h before harvest. To block CDK activity, embryos were treated with 25 μM PD0332991 (A8318, Palbociclib) or 25 μM CY202 (A1723, Palbociclib) from 18 hpf until harvest. To examine which pathway was required for protein degradation, HEK293T cells expressing either Flag-tagged zEtv2 or zScl were treated with 20 mM NH4Cl (A116363, Aladdin) or 20 μM MG132 (M7449, Sigma) for 5 h before harvest.

### BrdU incorporation

Two hours before embryos were fixed for BrdU incorporation experiments, they were incubated in 10 mM BrdU (B5002, Sigma-Aldrich) for 30 min at 4°C, then transferred to Holtfreter's solution to develop to desired stages at 28.5°C. ZsYellow proteins and incorporated BrdU were detected using the primary antibodies anti-ZsYellow (1:200; 632475, Clontech) and anti-BrdU (1:1000; B2531, Sigma-Aldrich), respectively.

### TUNEL assay

The TUNEL assay was performed using the In Situ Cell Death Detection Kit TMR red (12156792910, Roche) in accordance with the manufacturer's instructions.

### Cell lines and transfection

HEK293T (CRL-3216, ATCC) and HeLa (CCL-2, ATCC) cells were purchased from Shanghai Cell Bank and tested negative for mycoplasma contamination. These cell lines were authenticated using Short Tandem Repeat (STR) analysis by Shanghai Biowing Applied Biotechnology Company. HEK293T and HeLa cells were cultured in DMEM medium supplemented with 10% FBS in a 37°C humidified incubator. Cell transfections were conducted using Lipofectamine 2000 (11668019, Invitrogen) following the manufacturer's instructions.

### Immunoprecipitation assays

For co-immunoprecipitation assays, HEK293T cells were harvested and lysed with either TNE lysis buffer [10 mM Tris-HCl (pH 7.5), 150 mM NaCl, 2 mM EDTA and 0.5% Nonidet P-40] or RIPA buffer (R0010, Solarbio) containing a protease inhibitor mixture (1697498001, Roche). Lysates were incubated with anti-Flag-agarose beads (A2220, Sigma-Aldrich) or protein A-Sepharose beads (101041, Invitrogen) at 4°C for 4 h. Beads were washed four times with TNE or RIPA buffer, and bound proteins were then separated by SDS-PAGE and visualized using western blots.

For immunoblotting experiments, we used the following affinity-purified antibodies: anti-Flag (1:1000; Cell Signaling Technology, 2368S), anti-Myc (1:3000; M047-3, MBL), anti-HA (1:3000; CW0092A, CW), anti-β-tubulin (1:5000, CW0098M, CWBIO), anti-p-Thr (1:300; ab9337, Abcam) and anti-p-Ser (1:250; ab9332, Abcam).

### *In vitro* GST pull-down

GST fusion proteins were expressed in *E. coli* strain BL21 and purified using Glutathione-Sepharose 4B beads (71024800-GE, GE Healthcare). The purified GST-fusion proteins GST-zEtv2-(236-345) and GST-zScl were treated with thrombin (1:1000; T4648, Sigma) to cleave their GST tags. For *in vitro* binding assays, GST-AKT1 proteins were immobilized using Glutathione-Sepharose 4B beads and incubated with either purified zEtv2-(236-345) or zScl at 4°C for 3 h. After washing, bound proteins were separated with SDS-PAGE and analyzed using Coomassie Blue staining.

### *In vitro* kinase assay

For *in vitro* kinase assays, HEK293T cells were transfected with either Flag-tagged AKT1 or its kinase-deficient mutant. Then, 48 h after transfection, Flag-tagged proteins were enriched from cell extracts by immunoprecipitation and incubated with substrates that affinity purified *E. coli* in 1× reaction buffer [20 mM HEPES (pH 7.4), 10 mM MgCl_2_, 0.5 mM EGTA and 2 mM dithiothreitol) with 100 µM ATP (P0756S, New England Biolabs)] at 37°C for 30 min. The mixture was then separated on 10% SDS-PAGE and visualized either by western blot or Coomassie Blue staining.

### Statistical analysis

Student's *t*-test was used to analyze all data sets (Microsoft Excel). At a minimum, experiments were performed in triplicate. Results were considered statistically significant at *P*<0.05.

## Supplementary Material

10.1242/develop.200754_sup1 Supplementary information

## References

[DEV200754C1] Abrial, M., Paffett-Lugassy, N., Jeffrey, S., Jordan, D., O'Loughlin, E., Frederick, C. J., Burns, C. G. and Burns, C. E. (2017). TGF-β signaling is necessary and sufficient for pharyngeal arch artery angioblast formation. *Cell Rep.* 20, 973-983. 10.1016/j.celrep.2017.07.002 28746880 PMC5565225

[DEV200754C2] Anderson, M. J., Pham, V. N., Vogel, A. M., Weinstein, B. M. and Roman, B. L. (2008). Loss of unc45a precipitates arteriovenous shunting in the aortic arches. *Dev. Biol.* 318, 258-267. 10.1016/j.ydbio.2008.03.022 18462713 PMC2483962

[DEV200754C3] Ara, T., Tokoyoda, K., Okamoto, R., Koni, P. A. and Nagasawa, T. (2005). The role of CXCL12 in the organ-specific process of artery formation. *Blood* 105, 3155-3161. 10.1182/blood-2004-07-2563 15626744

[DEV200754C4] Bussmann, J., Wolfe, S. A. and Siekmann, A. F. (2011). Arterial-venous network formation during brain vascularization involves hemodynamic regulation of chemokine signaling. *Development* 138, 1717-1726. 10.1242/dev.059881 21429983 PMC3074448

[DEV200754C5] Cavallero, S., Shen, H., Yi, C., Lien, C.-L., Kumar, S. R. and Sucov, H. M. (2015). CXCL12 signaling is essential for maturation of the ventricular coronary endothelial plexus and establishment of functional coronary circulation. *Dev. Cell* 33, 469-477. 10.1016/j.devcel.2015.03.018 26017771 PMC4448078

[DEV200754C6] Charpentier, M. S. and Conlon, F. L. (2014). Cellular and molecular mechanisms underlying blood vessel lumen formation. *BioEssays* 36, 251-259. 10.1002/bies.201300133 24323945 PMC4187360

[DEV200754C7] Chen, J., Somanath, P. R., Razorenova, O., Chen, W. S., Hay, N., Bornstein, P. and Byzova, T. V. (2005). Akt1 regulates pathological angiogenesis, vascular maturation and permeability in vivo. *Nat. Med.* 11, 1188-1196. 10.1038/nm1307 16227992 PMC2277080

[DEV200754C8] Congdon, E. D. (1922). Transformation of the aortic-arch system during the development of the human embryo. *Contrib. Embryol.* 14, 49-47.

[DEV200754C9] De Azevedo, W. F., Leclerc, S., Meijer, L., Havlicek, L., Strnad, M. and Kim, S.-H. (1997). Inhibition of cyclin-dependent kinases by purine analogues: crystal structure of human cdk2 complexed with roscovitine. *Eur. J. Biochem.* 243, 518-526. 10.1111/j.1432-1033.1997.0518a.x 9030780

[DEV200754C10] Domanska, U. M., Kruizinga, R. C., Nagengast, W. B., Timmer-Bosscha, H., Huls, G., de Vries, E. G. E. and Walenkamp, A. M. E. (2013). A review on CXCR4/CXCL12 axis in oncology: no place to hide. *Eur. J. Cancer* 49, 219-230. 10.1016/j.ejca.2012.05.005 22683307

[DEV200754C11] Duda, D. G., Kozin, S. V., Kirkpatrick, N. D., Xu, L., Fukumura, D. and Jain, R. K. (2011). CXCL12 (SDF1α)-CXCR4/CXCR7 pathway inhibition: an emerging sensitizer for anticancer therapies? *Clin. Cancer Res.* 17, 2074-2080. 10.1158/1078-0432.CCR-10-2636 21349998 PMC3079023

[DEV200754C12] Dumon, N. A., Wang, Y. X. and Rudnicki, M. A. (2015). Intrinsic and extrinsic mechanisms regulating satellite cell function. *Development* 142, 1572-1581. 10.1242/dev.114223 25922523 PMC4419274

[DEV200754C13] Finley, D., Sadis, S., Monia, B. P., Boucher, P., Ecker, D. J., Crooke, S. T. and Chau, V. (1994). Inhibition of proteolysis and cell cycle progression in a multiubiquitination-deficient yeast mutant. *Mol. Cell. Biol.* 14, 5501-5509. 10.1128/mcb.14.8.5501-5509.1994 8035826 PMC359070

[DEV200754C14] Fry, D. W., Harvey, P. J., Keller, P. R., Elliott, W. L., Meade, M. A., Trachet, E., Albassam, M., Zheng, X. X., Leopold, W. R., Pryer, N. K. et al. (2004). Specific inhibition of cyclin-dependent kinase 4/6 by PD 0332991 and associated antitumor activity in human tumor xenografts. *Mol. Cancer Ther.* 3, 1427-1438. 10.1158/1535-7163.1427.3.11 15542782

[DEV200754C15] Fujita, M., Cha, Y. R., Pham, V. N., Sakurai, A., Roman, B. L., Gutkind, J. S. and Weinstein, B. M. (2011). Assembly and patterning of the vascular network of the vertebrate hindbrain. *Development* 138, 1705-1715. 10.1242/dev.058776 21429985 PMC3074447

[DEV200754C16] Guner-Ataman, B., González-Rosa, J. M., Shah, H. N., Butty, V. L., Jeffrey, S., Abrial, M., Boyer, L. A., Burns, C. G. and Burns, C. E. (2018). Failed progenitor specification underlies the cardiopharyngeal phenotypes in a zebrafish model of 22q11.2 deletion syndrome. *Cell Rep.* 24, 1342-1354.e1345. 10.1016/j.celrep.2018.06.117 30067987 PMC6261257

[DEV200754C17] Harrison, M. R. M., Bussmann, J., Huang, Y., Zhao, L., Osorio, A., Burns, C. G., Burns, C. E., Sucov, H. M., Siekmann, A. F. and Lien, C.-L. (2015). Chemokine-guided angiogenesis directs coronary vasculature formation in zebrafish. *Dev. Cell* 33, 442-454. 10.1016/j.devcel.2015.04.001 26017769 PMC4448080

[DEV200754C18] Hirai, H., Sootome, H., Nakatsuru, Y., Miyama, K., Taguchi, S., Tsujioka, K., Ueno, Y., Hatch, H., Majumder, P. K., Pan, B.-S. et al. (2010). MK-2206, an allosteric Akt inhibitor, enhances antitumor efficacy by standard chemotherapeutic agents or molecular targeted drugs in vitro and in vivo. *Mol. Cancer Ther.* 9, 1956-1967. 10.1158/1535-7163.MCT-09-1012 20571069

[DEV200754C19] Hiruma, T., Nakajima, Y. and Nakamura, H. (2002). Development of pharyngeal arch arteries in early mouse embryo. *J. Anat.* 201, 15-29. 10.1046/j.1469-7580.2002.00071.x 12171473 PMC1570898

[DEV200754C20] Hoffman, J. I. E. and Kaplan, S. (2002). The incidence of congenital heart disease. *J. Am. Coll. Cardiol.* 39, 1890-1900. 10.1016/S0735-1097(02)01886-7 12084585

[DEV200754C21] Hsieh, F.-C., Lu, Y.-F., Liau, I., Chen, C.-C., Cheng, C.-M., Hsiao, C.-D. and Hwang, S.-P. L. (2018). Zebrafish VCAP1X2 regulates cardiac contractility and proliferation of cardiomyocytes and epicardial cells. *Sci. Rep.* 8, 7856. 10.1038/s41598-018-26110-3 29777134 PMC5959901

[DEV200754C22] Ju, D. and Xie, Y. (2004). Proteasomal degradation of RPN4 via two distinct mechanisms, ubiquitin-dependent and -independent. *J. Biol. Chem.* 279, 23851-23854. 10.1074/jbc.C400111200 15090546

[DEV200754C23] Kadri, Z., Lefevre, C., Goupille, O., Penglong, T., Granger-Locatelli, M., Fucharoen, S., Maouche-Chretien, L., Leboulch, P. and Chretien, S. (2015). Erythropoietin and IGF-1 signaling synchronize cell proliferation and maturation during erythropoiesis. *Genes Dev.* 29, 2603-2616. 10.1101/gad.267633.115 26680303 PMC4699388

[DEV200754C24] Kameda, Y. (2009). Hoxa3 and signaling molecules involved in aortic arch patterning and remodeling. *Cell Tissue Res.* 336, 165-178. 10.1007/s00441-009-0760-7 19290546

[DEV200754C25] Katsumoto, K. and Kume, S. (2011). Endoderm and mesoderm reciprocal signaling mediated by CXCL12 and CXCR4 regulates the migration of angioblasts and establishes the pancreatic fate. *Development* 138, 1947-1955. 10.1242/dev.058719 21490062

[DEV200754C26] Kodo, K. and Yamagishi, H. (2011). A decade of advances in the molecular embryology and genetics underlying congenital heart defects. *Circ. J.* 75, 2296-2304. 10.1253/circj.CJ-11-0636 21914956

[DEV200754C27] Lampugnani, M. G., Orsenigo, F., Rudini, N., Maddaluno, L., Boulday, G., Chapon, F. and Dejana, E. (2010). CCM1 regulates vascular-lumen organization by inducing endothelial polarity. *J. Cell Sci.* 123, 1073-1080. 10.1242/jcs.059329 20332120

[DEV200754C28] Lee, M. Y., Luciano, A. K., Ackah, E., Rodriguez-Vita, J., Bancroft, T. A., Eichmann, A., Simons, M., Kyriakides, T. R., Morales-Ruiz, M. and Sessa, W. C. (2014). Endothelial Akt1 mediates angiogenesis by phosphorylating multiple angiogenic substrates. *Proc. Natl. Acad. Sci. USA* 111, 12865-12870. 10.1073/pnas.1408472111 25136137 PMC4156707

[DEV200754C29] Li, L. W., Ning, G. Z., Yang, S. Y., Yan, Y. F., Cao, Y. and Wang, Q. (2019). BMP signaling is required for nkx2.3-positive pharyngeal pouch progenitor specification in zebrafish. *PLoS Genet.* 15, e1007996. 10.1371/journal.pgen.1007996 30763319 PMC6392332

[DEV200754C30] Liang, J., Zubovitz, J., Petrocelli, T., Kotchetkov, R., Connor, M. K., Han, K., Lee, J.-H., Ciarallo, S., Catzavelos, C., Beniston, R. et al. (2002). PKB/Akt phosphorylates p27, impairs nuclear import of p27 and opposes p27-mediated G1 arrest. *Nat. Med.* 8, 1153-1160. 10.1038/nm761 12244302

[DEV200754C31] Liu, C., Li, Y., Semenov, M., Han, C., Baeg, G.-H., Tan, Y., Zhang, Z., Lin, X. and He, X. (2002). Control of beta-catenin phosphorylation/degradation by a dual-kinase mechanism. *Cell* 108, 837-847. 10.1016/S0092-8674(02)00685-2 11955436

[DEV200754C32] Liu, J., Zhu, C., Ning, G., Yang, L., Cao, Y., Huang, S. and Wang, Q. (2019). Chemokine signaling links cell-cycle progression and cilia formation for left-right symmetry breaking. *PLoS Biol.* 17, e3000203. 10.1371/journal.pbio.3000203 31430272 PMC6716676

[DEV200754C33] Manning, B. D. and Toker, A. (2017). AKT/PKB signaling: navigating the network. *Cell* 169, 381-405. 10.1016/j.cell.2017.04.001 28431241 PMC5546324

[DEV200754C34] Mao, A., Zhang, M., Liu, J., Cao, Y. and Wang, Q. (2019). PDGF signaling from pharyngeal pouches promotes arch artery morphogenesis. *J. Genet. Genomics* 46, 551-559. 10.1016/j.jgg.2019.11.004 31974005

[DEV200754C35] Mao, A., Zhang, M., Li, L., Liu, J., Ning, G., Cao, Y. and Wang, Q. (2021). Pharyngeal pouches provide a niche microenvironment for arch artery progenitor specification. *Development* 148, dev192658. 10.1242/dev.192658 33334861 PMC7847271

[DEV200754C36] Meng, Z.-Z., Liu, W., Xia, Y., Yin, H.-M., Zhang, C.-Y., Su, D., Yan, L.-F., Gu, A.-H. and Zhou, Y. (2017). The pro-inflammatory signalling regulator Stat4 promotes vasculogenesis of great vessels derived from endothelial precursors. *Nat. Commun.* 8, 14640. 10.1038/ncomms14640 28256502 PMC5338034

[DEV200754C37] Montero-Balaguer, M., Swirsding, K., Orsenigo, F., Cotelli, F., Mione, M. and Dejana, E. (2009). Stable vascular connections and remodeling require full expression of VE-cadherin in zebrafish embryos. *PLoS ONE* 4, e5772. 10.1371/journal.pone.0005772 19503615 PMC2685470

[DEV200754C38] Muratani, M. and Tansey, W. P. (2003). How the ubiquitin-proteasome system controls transcription. *Nat. Rev. Mol. Cell Biol.* 4, 192-201. 10.1038/nrm1049 12612638

[DEV200754C39] Nagelberg, D., Wang, J., Su, R., Torres-Vázquez, J., Targoff, K. L., Poss, K. D. and Knaut, H. (2015). Origin, specification, and plasticity of the great vessels of the heart. *Curr. Biol.* 25, 2099-2110. 10.1016/j.cub.2015.06.076 26255850 PMC4546555

[DEV200754C40] Nair, S. and Schilling, T. F. (2008). Chemokine signaling controls endodermal migration during zebrafish gastrulation. *Science* 322, 89-92. 10.1126/science.1160038 18719251 PMC2770598

[DEV200754C41] Nakajima, K., Inagawa, M., Uchida, C., Okada, K., Tane, S., Kojima, M., Kubota, M., Noda, M., Ogawa, S., Shirato, H. et al. (2011). Coordinated regulation of differentiation and proliferation of embryonic cardiomyocytes by a jumonji (Jarid2)-cyclin D1 pathway. *Development* 138, 1771-1782. 10.1242/dev.059295 21447557

[DEV200754C42] Nakajo, N., Deno, Y.-K., Ueno, H., Kenmochi, C., Shimuta, K. and Sagata, N. (2011). Temporal and spatial expression patterns of Cdc25 phosphatase isoforms during early Xenopus development. *Int. J. Dev. Biol.* 55, 627-632. 10.1387/ijdb.113287nn 21948711

[DEV200754C43] Nicoli, S., Standley, C., Walker, P., Hurlstone, A., Fogarty, K. E. and Lawson, N. D. (2010). MicroRNA-mediated integration of haemodynamics and Vegf signalling during angiogenesis. *Nature* 464, 1196-1200. 10.1038/nature08889 20364122 PMC2914488

[DEV200754C44] Ning, G., Liu, X., Dai, M., Meng, A. and Wang, Q. (2013). MicroRNA-92a upholds Bmp signaling by targeting noggin3 during pharyngeal cartilage formation. *Dev. Cell* 24, 283-295. 10.1016/j.devcel.2012.12.016 23410941

[DEV200754C45] Olesnicky Killian, E. C., Birkholz, D. A. and Artinger, K. B. (2009). A role for chemokine signaling in neural crest cell migration and craniofacial development. *Dev. Biol.* 333, 161-172. 10.1016/j.ydbio.2009.06.031 19576198 PMC2728170

[DEV200754C46] Paffett-Lugassy, N., Singh, R., Nevis, K. R., Guner-Ataman, B., O'Loughlin, E., Jahangiri, L., Harvey, R. P., Burns, C. G. and Burns, C. E. (2013). Heart field origin of great vessel precursors relies on nkx2.5-mediated vasculogenesis. *Nat. Cell Biol.* 15, 1362-1225. 10.1038/ncb2862 24161929 PMC3864813

[DEV200754C47] Paffett-Lugassy, N., Novikov, N., Jeffrey, S., Abrial, M., Guner-Ataman, B., Sakthivel, S., Burns, C. E. and Burns, C. G. (2017). Unique developmental trajectories and genetic regulation of ventricular and outflow tract progenitors in the zebrafish second heart field. *Development* 144, 4616-4624. 10.1242/dev.153411 29061637 PMC5769620

[DEV200754C48] Pauklin, S. and Vallier, L. (2013). The cell-cycle state of stem cells determines cell fate propensity. *Cell* 155, 135-147. 10.1016/j.cell.2013.08.031 24074866 PMC3898746

[DEV200754C49] Psillas, G., Kekes, G., Constantinidis, J., Triaridis, S. and Vital, V. (2007). Subclavian steal syndrome: neurotological manifestations. *Acta Otorhinolaryngol. Ital.* 27, 33-37. 17601209 PMC2640015

[DEV200754C50] Rana, M. S., Sizarov, A., Christoffels, V. M. and Moorman, A. F. M. (2014). Development of the human aortic arch system captured in an interactive three-dimensional reference model. *Am. J. Med. Genet. A* 164A, 1372-1383. 10.1002/ajmg.a.35881 23613216

[DEV200754C51] Raz, E. and Mahabaleshwar, H. (2009). Chemokine signaling in embryonic cell migration: a fisheye view. *Development* 136, 1223-1229. 10.1242/dev.022418 19304885

[DEV200754C52] Ren, X., Gomez, G. A., Zhang, B. and Lin, S. (2010). Scl isoforms act downstream of etsrp to specify angioblasts and definitive hematopoietic stem cells. *Blood* 115, 5338-5346. 10.1182/blood-2009-09-244640 20185582 PMC2902133

[DEV200754C53] Rossi, A., Kontarakis, Z., Gerri, C., Nolte, H., Hölper, S., Krüger, M. and Stainier, D. Y. R. (2015). Genetic compensation induced by deleterious mutations but not gene knockdowns. *Nature* 524, 230-233. 10.1038/nature14580 26168398

[DEV200754C54] Sanidas, I., Polytarchou, C., Hatziapostolou, M., Ezell, S. A., Kottakis, F., Hu, L., Guo, A., Xie, J., Comb, M. J., Iliopoulos, D. et al. (2014). Phosphoproteomics screen reveals akt isoform-specific signals linking RNA processing to lung cancer. *Mol. Cell* 53, 577-590. 10.1016/j.molcel.2013.12.018 24462114 PMC3947584

[DEV200754C55] Shan, J., Nguyen, T. B., Totary-Jain, H., Dansky, H., Marx, S. O. and Marks, A. R. (2008). Leptin-enhanced neointimal hyperplasia is reduced by mTOR and PI3K inhibitors. *Proc. Natl. Acad. Sci. USA* 105, 19006-19011. 10.1073/pnas.0809743105 19020099 PMC2585045

[DEV200754C56] Sherr, C. J. and Roberts, J. M. (1999). CDK inhibitors: positive and negative regulators of G1-phase progression. *Genes Dev.* 13, 1501-1512. 10.1101/gad.13.12.1501 10385618

[DEV200754C57] Siekmann, A. F., Standley, C., Fogarty, K. E., Wolfe, S. A. and Lawson, N. D. (2009). Chemokine signaling guides regional patterning of the first embryonic artery. *Genes Dev.* 23, 2272-2277. 10.1101/gad.1813509 19797767 PMC2758748

[DEV200754C58] Sincennes, M.-C., Humbert, M., Grondin, B., Lisi, V., Veiga, D. F. T., Haman, A., Cazaux, C., Mashtalir, N., Affar, E. L. B., Verreault, A. et al. (2016). The LMO2 oncogene regulates DNA replication in hematopoietic cells. *Proc. Natl. Acad. Sci. USA* 113, 1393-1398. 10.1073/pnas.1515071113 26764384 PMC4747768

[DEV200754C59] Stückemann, T., Wegleiter, T., Stefan, E., Nägele, O., Tarbashevich, K., Böck, G., Raz, E. and Aanstad, P. (2012). Zebrafish Cxcr4a determines the proliferative response to Hedgehog signalling. *Development* 139, 2711-2720. 10.1242/dev.074930 22782722

[DEV200754C60] Su, T. Y., Stanley, G., Sinha, R., D'Amato, G., Das, S., Rhee, S., Chang, A. H., Poduri, A., Raftrey, B., Dinh, T. T. et al. (2018). Single-cell analysis of early progenitor cells that build coronary arteries. *Nature* 559, 356-362. 10.1038/s41586-018-0288-7 29973725 PMC6053322

[DEV200754C61] Sugiyama, M., Sakaue-Sawano, A., Iimura, T., Fukami, K., Kitaguchi, T., Kawakami, K., Okamoto, H., Higashijima, S. and Miyawaki, A. (2009). Illuminating cell-cycle progression in the developing zebrafish embryo. *Proc. Natl. Acad. Sci. USA* 106, 20812-20817. 10.1073/pnas.0906464106 19923430 PMC2779202

[DEV200754C62] Sumanas, S. and Lin, S. (2006). Ets1-related protein is a key regulator of vasculogenesis in zebrafish. *PLoS Biol.* 4, 60-69. 10.1371/journal.pbio.0040010 PMC1310653 16336046

[DEV200754C63] Tachibana, K., Hirota, S., Iizasa, H., Yoshida, H., Kawabata, K., Kataoka, Y., Kitamura, Y., Matsushima, K., Yoshida, N., Nishikawa, S.-I. et al. (1998). The chemokine receptor CXCR4 is essential for vascularization of the gastrointestinal tract. *Nature* 393, 591-594. 10.1038/31261 9634237

[DEV200754C64] Takabatake, Y., Sugiyama, T., Kohara, H., Matsusaka, T., Kurihara, H., Koni, P. A., Nagasawa, Y., Hamano, T., Matsui, I., Kawada, N. et al. (2009). The CXCL12 (SDF-1)/CXCR4 axis is essential for the development of renal vasculature. *J. Am. Soc. Nephrol.* 20, 1714-1723. 10.1681/ASN.2008060640 19443644 PMC2723985

[DEV200754C65] Terme, J.-M., Lhermitte, L., Asnafi, V. and Jalinot, P. (2009). TGF-β induces degradation of TAL1/SCL by the ubiquitin-proteasome pathway through AKT-mediated phosphorylation. *Blood* 113, 6695-6698. 10.1182/blood-2008-07-166835 19406989

[DEV200754C66] Viglietto, G., Motti, M. L., Bruni, P., Melillo, R. M., D'Alessio, A., Califano, D., Vinci, F., Chiappetta, G., Tsichlis, P., Bellacosa, A. et al. (2002). Cytoplasmic relocalization and inhibition of the cyclin-dependent kinase inhibitor p27(Kip1) by PKB/Akt-mediated phosphorylation in breast cancer. *Nat. Med.* 8, 1136-1144. 10.1038/nm762 12244303

[DEV200754C67] Wang, X., Chen, D., Chen, K., Jubran, A., Ramirez, A. J. and Astrof, S. (2017). Endothelium in the pharyngeal arches 3, 4 and 6 is derived from the second heart field. *Dev. Biol.* 421, 108-117. 10.1016/j.ydbio.2016.12.010 27955943 PMC5221477

[DEV200754C68] Wei, S., Dai, M. M., Liu, Z. T., Ma, Y. Q., Shang, H. Q., Cao, Y. and Wang, Q. (2017). The guanine nucleotide exchange factor Net1 facilitates the specification of dorsal cell fates in zebrafish embryos by promoting maternal β-catenin activation. *Cell Res.* 27, 202-225. 10.1038/cr.2016.141 27910850 PMC5339846

[DEV200754C69] Widberg, C. H., Newell, F. S., Bachmann, A. W., Ramnoruth, S. N., Spelta, M. C., Whitehead, J. P., Hutley, L. J. and Prins, J. B. (2009). Fibroblast growth factor receptor 1 is a key regulator of early adipogenic events in human preadipocytes. *Am. J. Physiol. Endocrinol. Metabol.* 296, E121-E131. 10.1152/ajpendo.90602.2008 18940940

[DEV200754C70] Wolman, M. A., Jain, R. A., Marsden, K. C., Bell, H., Skinner, J., Hayer, K. E., Hogenesch, J. B. and Granato, M. (2015). A genome-wide screen identifies PAPP-AA-mediated IGFR signaling as a novel regulator of habituation learning (vol 85, pg 1200, 2015). *Neuron* 87, 906-907. 10.1016/j.neuron.2015.08.009 PMC4368495 25754827

[DEV200754C71] Zhou, B. P., Liao, Y., Xia, W., Spohn, B., Lee, M.-H. and Hung, M.-C. (2001). Cytoplasmic localization of p21Cip1/WAF1 by Akt-induced phosphorylation in HER-2/neu-overexpressing cells. *Nat. Cell Biol.* 3, 245-252. 10.1038/35060032 11231573

