# Supplementary material for: Chemokine signaling synchronizes angioblast proliferation and differentiation during pharyngeal arch artery vasculogenesis
Source: Development. 2022 Dec 5;149(23):dev200754. doi: 10.1242/dev.200754 (PMC10114070; doi:10.1242/dev.200754)
Supplement: Supplementary information [file develop-149-200754-s1.pdf]

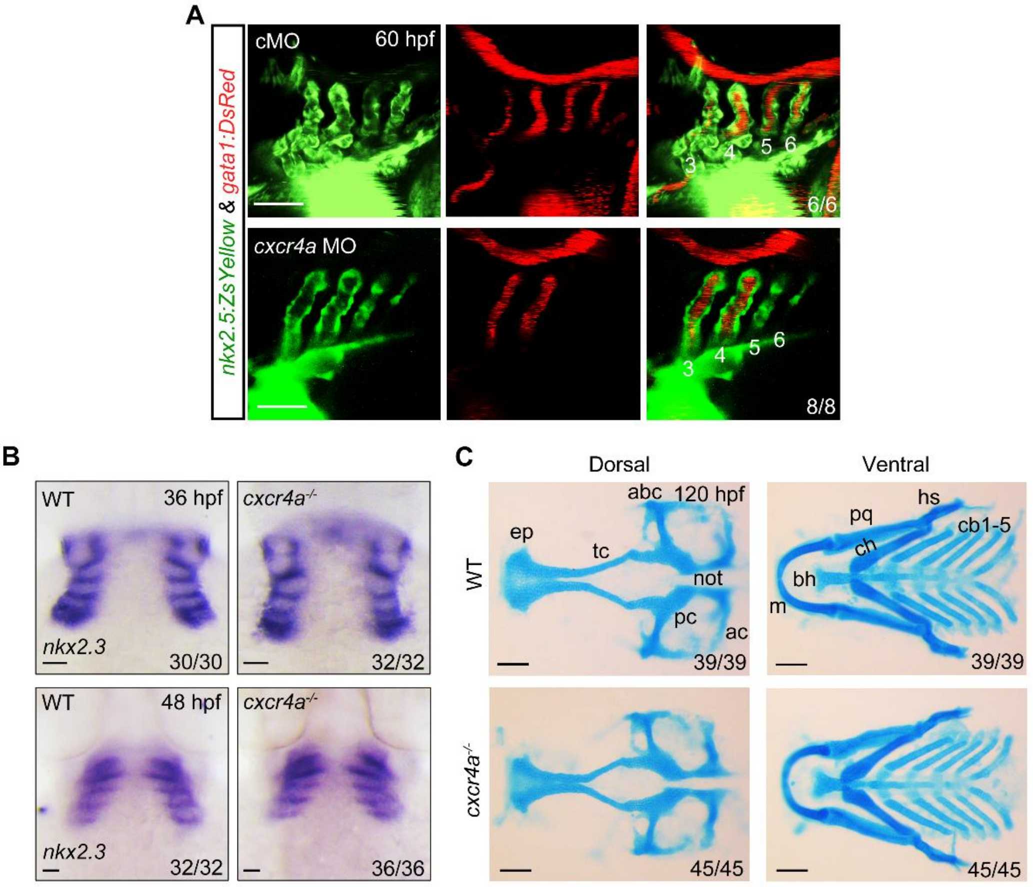

Fig. S1. The pharyngeal pouches and craniofacial cartilages develop normally in *cxcr4a*$^{-/-}$ mutants. (A) *cxcr4a* morphants displayed vascular stenosis in the posterior PAAs. Representative confocal images of embryos injected with 4 ng control MO (cMO) or *cxcr4a* MO are shown. The ratios of affected embryos are indicated in the lower right corner. Scale bars, 50 µm. (B) Expression analysis of the pharyngeal pouch marker gene *nkx2.3* in wild-type (WT) and *cxcr4a*$^{-/-}$ embryos by in *situ* hybridizations at 36 hpf and 48 hpf. Scale bars, 50 µm. (C) Alcian Blue staining of craniofacial cartilages in wild-type (WT) and *cxcr4a*$^{-/-}$ embryos at 120 hpf. ep, ep, ethmoid plate; tc, trabeculae cranii; pc, parachordal; ac, auditory capsule; abc, anterior basicranial commissure; m, Meckel's cartilage; not, notochord; hs, hyosymplectic; pq, palatoquadrate; bh, basihyal; ch, ceratohyal; cb, ceratobranchial. Scale bars, 200µm.

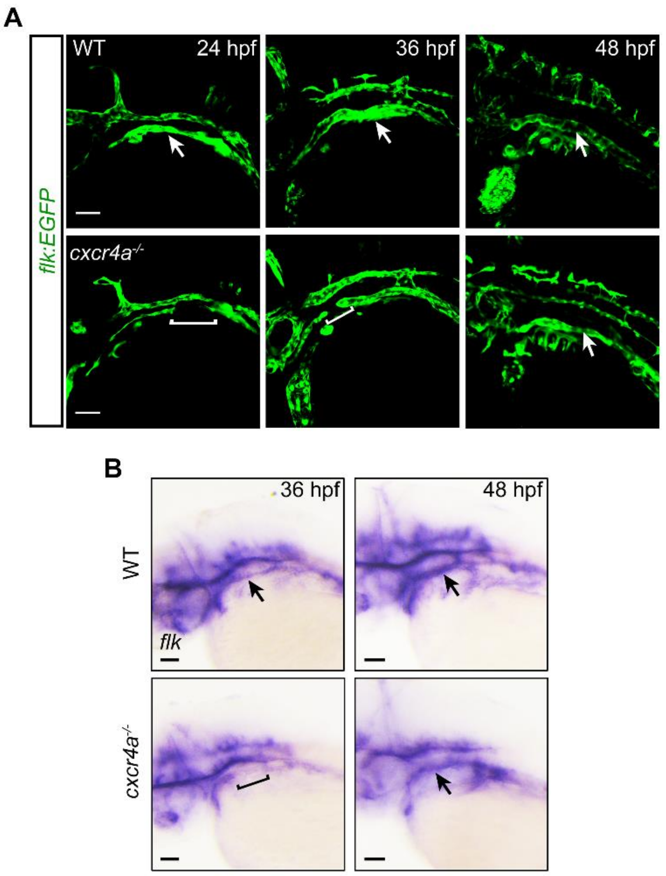

Fig. S2. The defect in LDA formation in cxcr4a-/- mutants is gradually restored. (A) Representative confocal images of LDA in wild-type (WT) and *cxcr4a*$^{-/-}$ embryos at the indicated stages. White arrows indicate the LDA, and the brackets indicate the gap within LDA. Scale bars, 50 µm. (B) The expression of *flk* was examined by in *situ* hybridization in wild-type (WT) and *cxcr4a*$^{-/-}$ embryos at 36 hpf and 48 hpf. Black arrows indicate the LDA, and the bracket indicates the gap within LDA. Scale bars, 50µm.

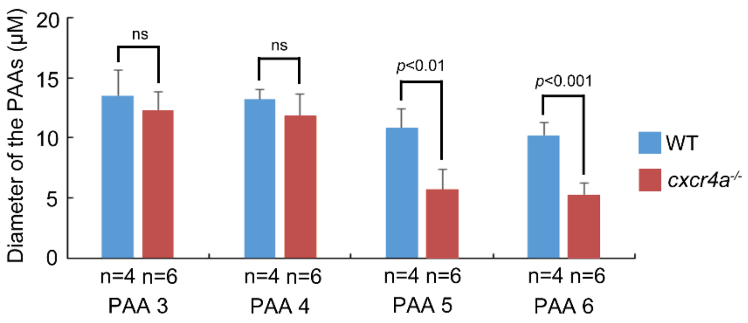

Fig. S3. The diameter of PAAs 5-6 is obviously reduced in *cxcr4a*$^{-/-}$ mutants. The diameters of PAAs in wild-type (WT) and *cxcr4a*-deficient *Tg(fli1:nucEGFP)* embryos at 60 hpf were measured at intermediate position along each PAA using the line tool in Image J. Error bars indicate the standard deviation of three biological replicates. Student's *t*-test, ns, no significant.

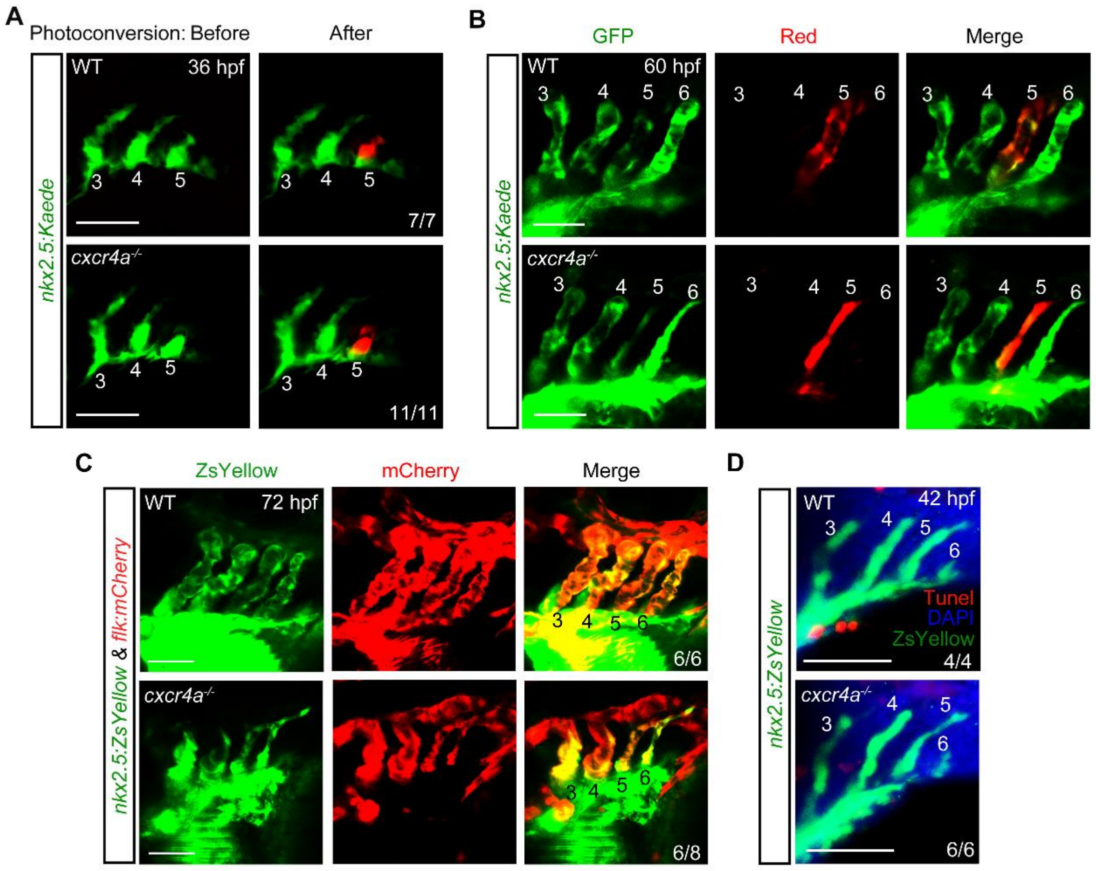

Fig. S4. Lack of *cxcr4a* has no influence in PAA angioblast migration and survival. (A) The PAA cluster 5 in wild-type (WT) or *cxcr4a*-depleted *Tg(nkx2.5:Kaede)* embryos was photoconverted at 36 hpf. Scale bars, 50 µm. (B) The resulting embryos were imaged at 60 hpf. Scale bars, 50 µm. (C) The PAA 5 and PAA6 in *cxcr4a*$^{-/-}$ mutants were successfully fused and connected with LDA. Live confocal images of wild-type (WT) and *cxcr4a*$^{-/-}$ mutants on the *Tg(nkx2.5:ZsYellow;flk:mCherry)* background. Scale bars, 50 µm. (D) Genetic depletion of *cxcr4a* does not lead to apoptosis in PAA angioblasts. Apoptotic cells (red) in wild-type (WT) and *cxcr4a*$^{-/-}$ embryos expressing *nkx2.5:ZsYellow* transgene (green) were detected by TUNEL assays at 42 hpf. Scale bars, 50 µm.

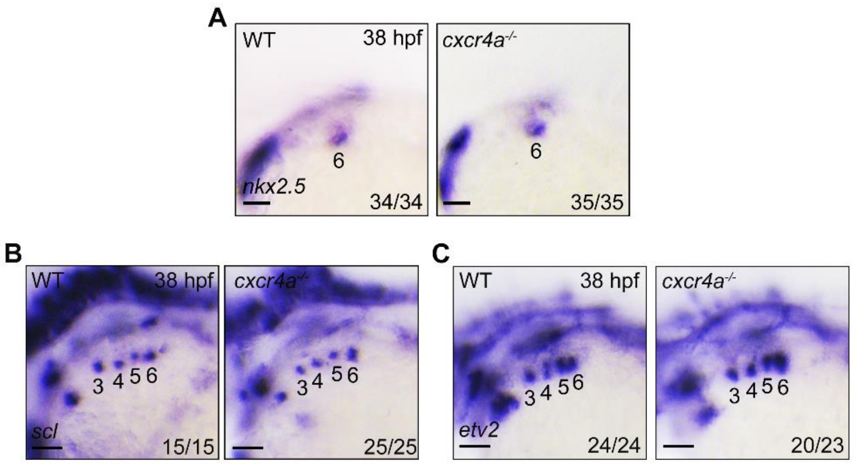

Fig. S5. *cxcr4a* is not required for PAA progenitor specification and angioblast transition. (A-C) Expression analysis of *nkx2.5* (A), *scl* (B) and *etv2* (C) in wild-type (WT) control and *cxcr4a*$^{-/-}$ mutants by in situ hybridization. All the Scale bars are 50 µm.

Development • Supplementary information

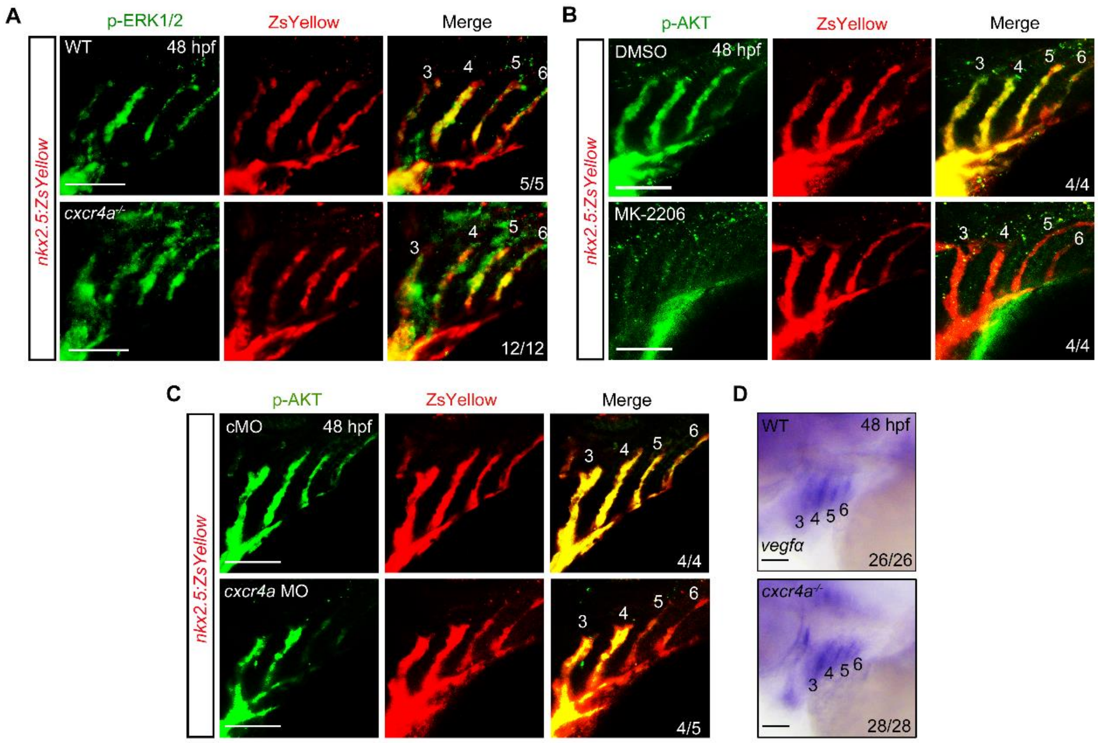

Fig. S6. Inactivity of *cxcr4a* does not affect ERK1/2 phosphorylation and *vegfa* expression, but reduces p-AKT expression levels in the posterior PAAs. (A) Wild-type (WT) and *cxcr4a*-deficient *Tg(nkx2.5:ZsYellow)* embryos were harvested at 48 hpf and subjected to immunostaining for p-ERK1/2 (green) and ZsYellow (red). Scale bars, 50 µm. (B) *Tg(nkx2.5:ZsYellow)* embryos treated with DMSO or AKT inhibitor MK-2206 from 18 hpf were immunostained for ZsYellow (red) and p-AKT (green). Scale bars, 50 µm. (C) *Tg(nkx2.5:ZsYellow)* embryos were injected with 4 ng control MO (cMO) or *cxcr4a* MO at one-cell stage, and then immunostained for p-AKT (green) and ZsYellow (red) at 48 hpf. Scale bars, 50 µm. (D) Expression analysis of *vegfa* transcripts in wild-type (WT) and *cxcr4a*$^{-/-}$ embryos by in situ hybridization. Scale bars, 50 µm.

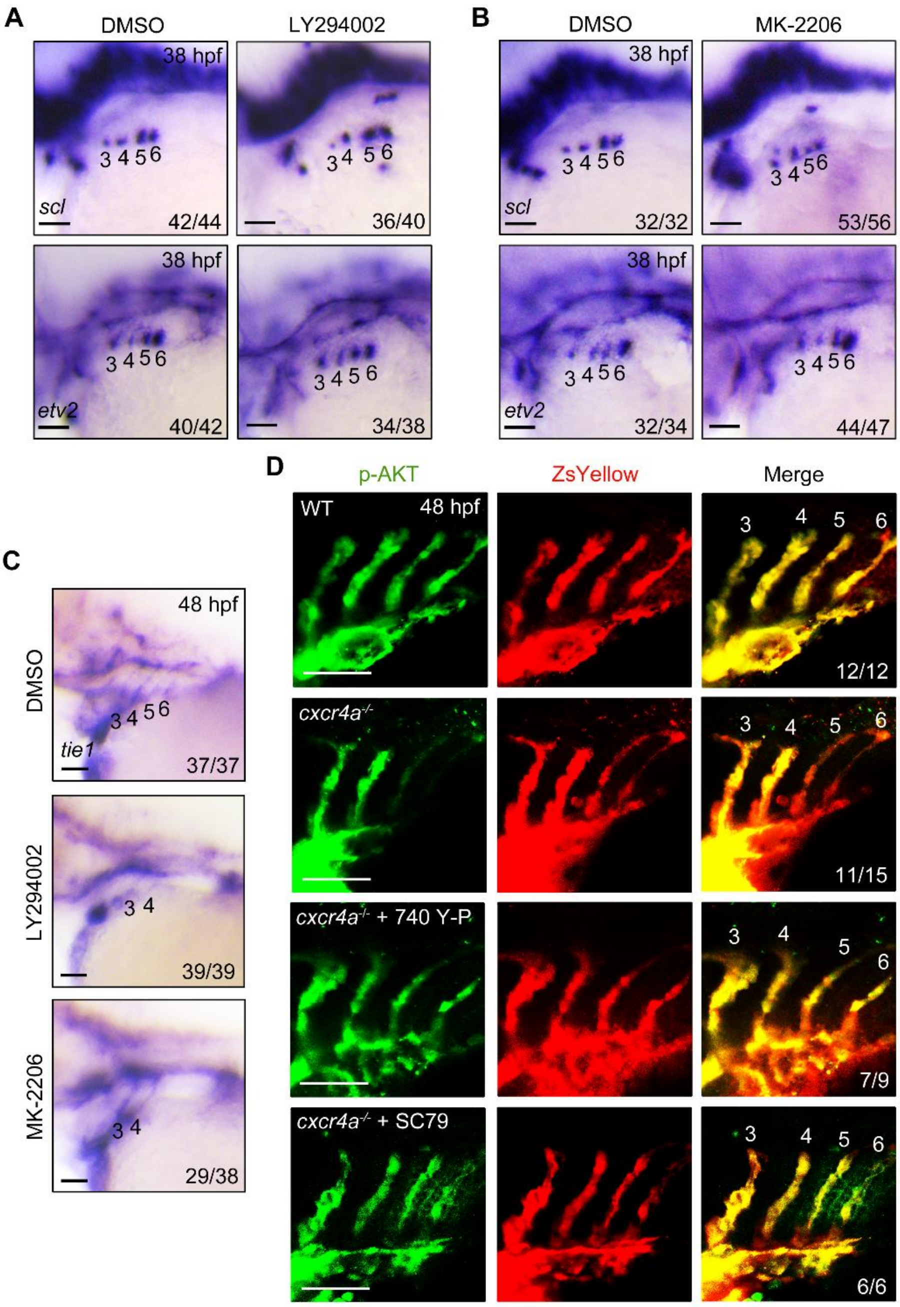

Fig. S7. PI3K/AKT signaling is not involved in PAA progenitor-to-angioblast transition, but is required for PAA angioblast differentiation. (A-B) The expression of *scl* and *etv2* were evaluated by *in situ* hybridization in either DMSO or PI3K/AKT inhibitor-treated embryos at 38 hpf. Embryos were treated with either DMSO or inhibitors from 18 to 38 hpf. PAA angioblast clusters are indicated with corresponding numbers. LY294002, PI3K inhibitor. MK-2206, AKT inhibitor. Scale bars, 50 µm. (C) Analysis of *tie1* expression in wild-type embryos treated with DMSO or PI3K inhibitor LY294002 or AKT inhibitor MK-2206 from 36 to 48 hpf. Scale bars, 50 µm. (D) PI3K agonist 740 Y-P or AKT agonist SC79 treatment could restore the reduced p-AKT levels in the posterior PAAs of *cxcr4a*$^{-/-}$ mutants. Wild-type (WT) and *cxcr4a*$^{-/-}$ mutant embryos on the *Tg(nkx2.5:ZsYellow)* background treated with or without indicated agonist were immunostained with anti-p-AKT (green) and anti-ZsYellow (red) antibodies. Scale bars, 50 µm.

Development • Supplementary information

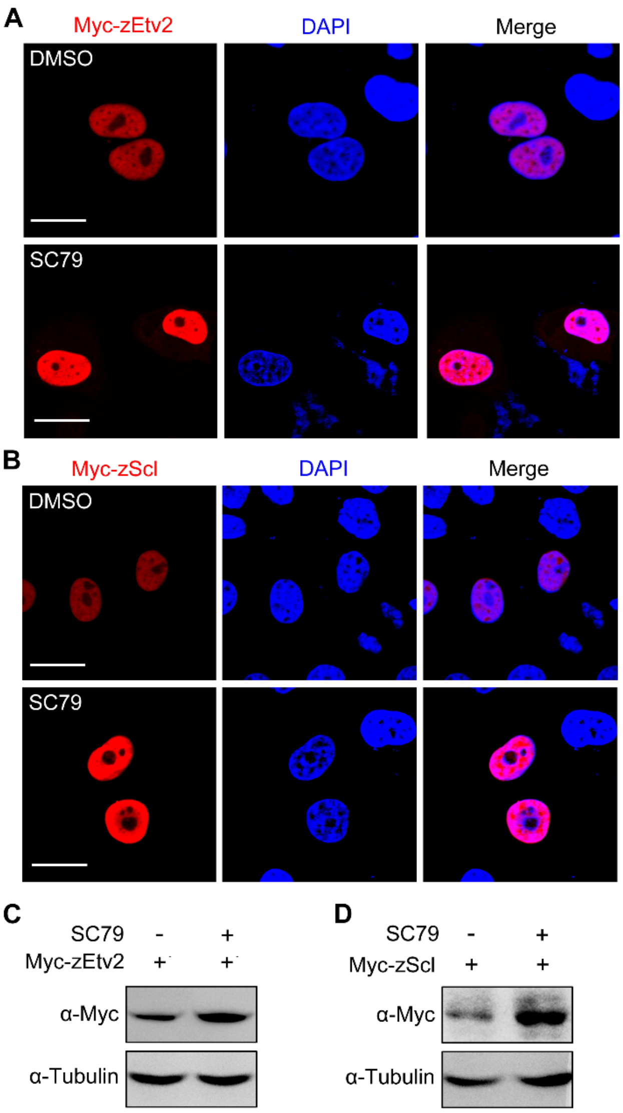

Fig. S8. Activation of AKT signaling increases the protein expression of both zEtv2 and zScl. (A-B) HeLa cells transfected with either Myc-zEtv2 or Myc-zScl were treated with DMSO or 10 µM SC79 and immunostained with an anti-Myc (red) antibody and the nuclear stain DAPI (blue). Scale bars, 20 µm. (C-D) HEK293T cells were transfected with Myc-tagged zEtv2 or zScl and then harvested for Western blot after being treated with DMSO or 10 µM SC79.

Development • Supplementary information

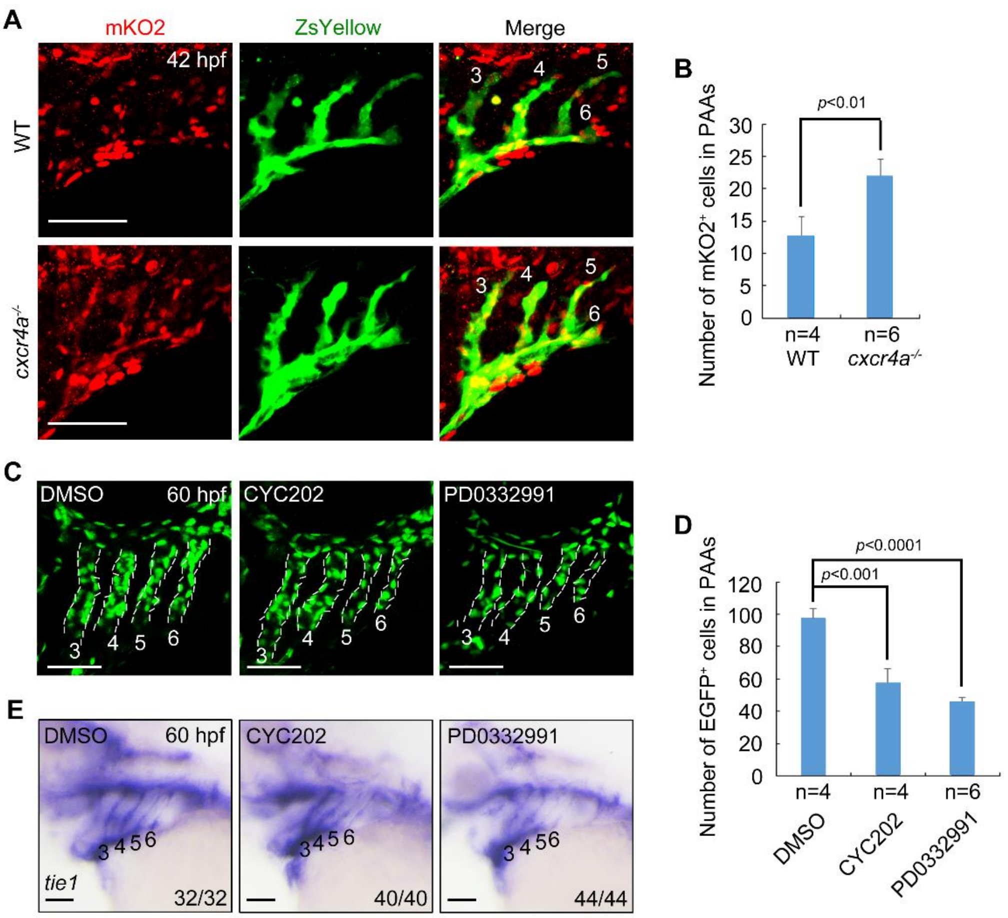

Fig. S9. G1 phase CDKs are unnecessary for PAA angioblast differentiation. (A-B) *cxcr4a* deficiency inhibits the G1/S transition in PAAs. Wild-type (WT) and *cxcr4a*-deficient *Tg(nkx2.5:ZsYellow;EF1α:mKO2-zCdt1(1/190))* embryos at 42 hpf were co-immunostained with anti-mKO2 (red) and anti-ZsYellow (green) antibodies. Scale bars, 50 µm. The numbers of mKO2^{+} cells in PAAs were quantified and shown in (B). Error bars indicate standard deviation of three biological replicates. (C-D) PAAs of *Tg(fli1:nucEGFP)* embryos treated with 25 µM CYC202 or PD0332991 were imaged and representative images are shown in (C). Scale bars, 50 µm. The numbers of EGFP^{+} cells in PAAs were quantified and described in (D). Error bars indicate standard deviation of three biological replicates. CYC202, CDK2 inhibitor. PD0332991, CDK4/6 inhibitor. (E) Expression analysis of *tie1* in 25 µM CYC202 or PD0332991 treated-embryos at 60 hpf by *in situ* hybridization. Scale bars, 50 µm.

Development • Supplementary information

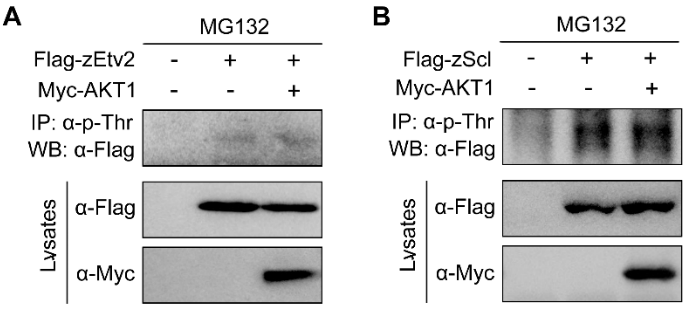

Fig. S10. The phospho-threonine levels of zEtv2 and zScl were unaffected by AKT1 overexpression. (A-B) HEK293T cells were transfected with the indicated plasmids and were lysed to immunoprecipitate using an anti-p-threonine antibody and then blotted with anti-Flag antibody to detect phosphorylated zEtv2 or zScl.

Development • Supplementary information

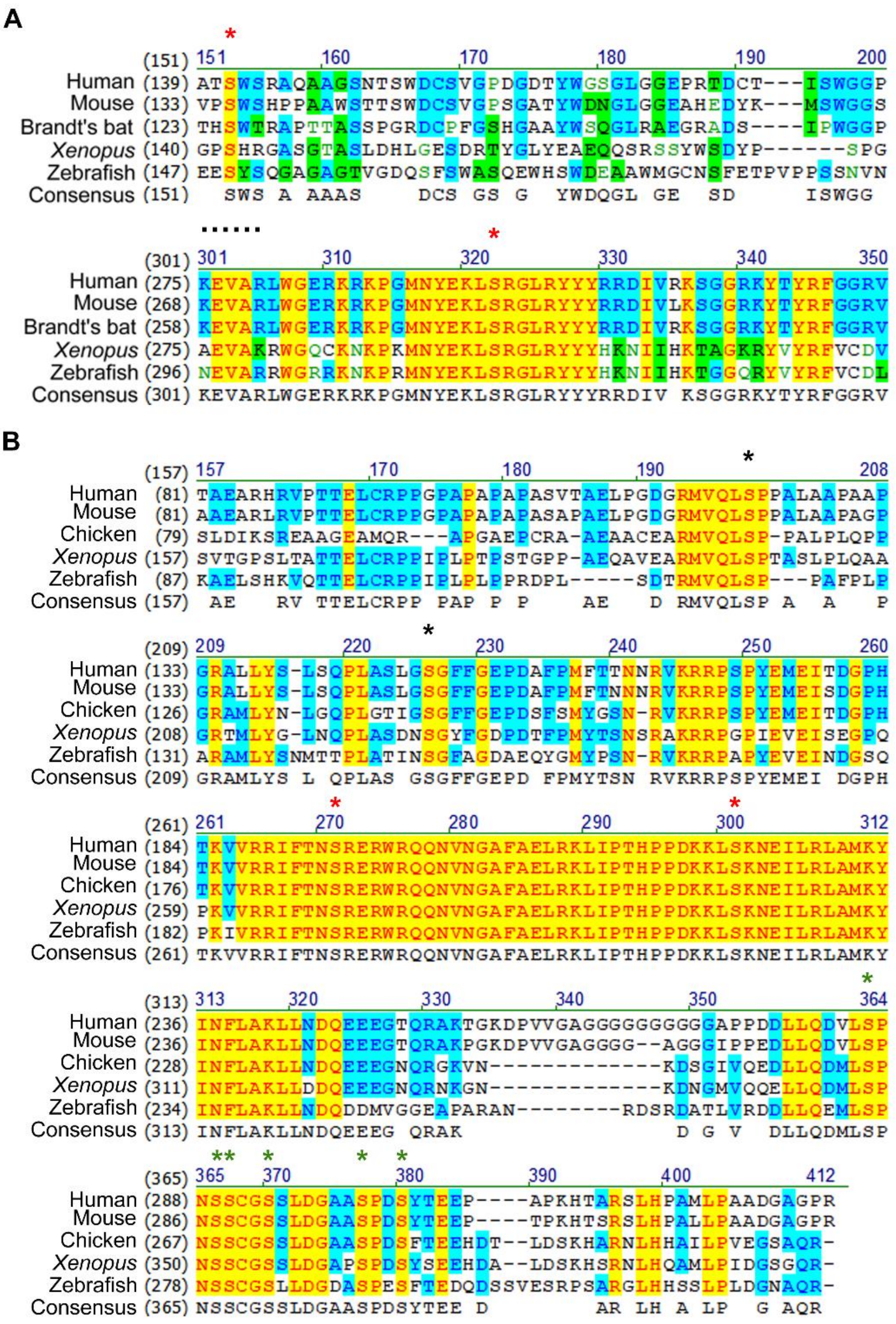

Fig. S11. Conservative serine sites within zEtv2 and zScl proteins. (A) Protein sequence alignment of zEtv2 with the homologous proteins. Conservative serine sites are indicated by red asterisks. (B) Conservative serine sites of zScl are divided into three groups indicated by black (SI group), red (SII group), and green (SIII group) asterisks.

Development • Supplementary information

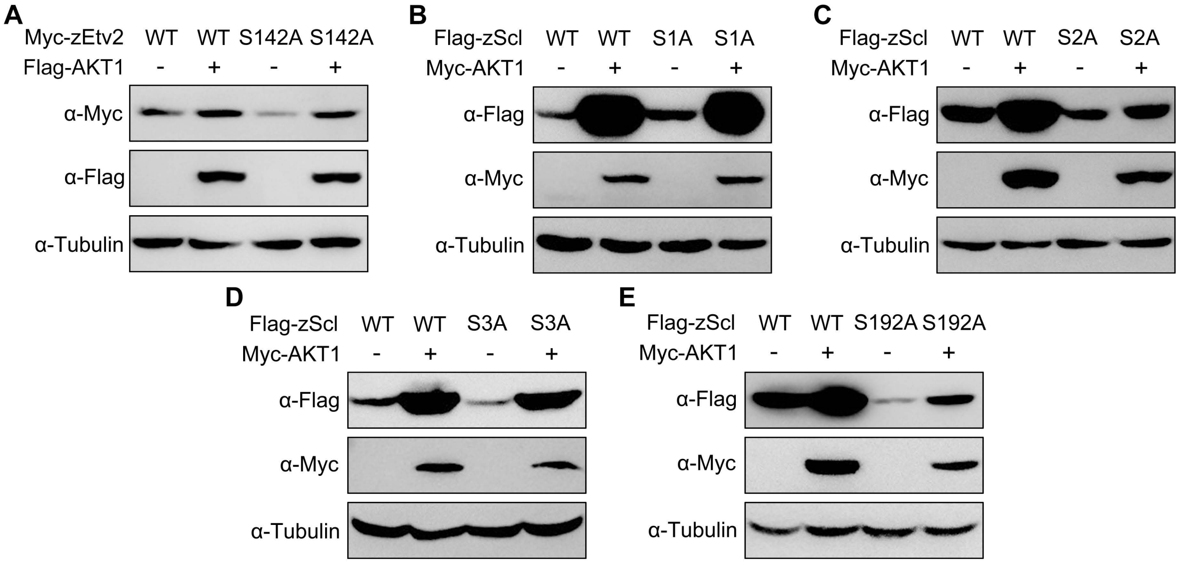

Fig. S12. AKT differently regulates the protein stability of various S-A mutants of zEtv2 and zScl. (A) The zEtv2-S142A mutant is stabilized by AKT1 overexpression. HEK293T cells were transfected with the indicated plasmids and harvested for Western blot analysis. The lysate was then probed with anti-Flag, anti-Myc and anti-Tubulin antibodies. (B-E) HEK293T cells transfected with the indicated constructs were lysed for Western blot analysis using the indicated antibodies. The three groups of conservative serine sites in zScl (Fig. S11B) were mutated to alanine (B-D). Note that when the serine residues (192 and 222) of the SII group were substituted with alanine, the resulting mutant lose its response to AKT1 overexpression (C). Overexpression of AKT1 remained able to stabilize zScl-S192A mutant (E).

Development • Supplementary information
