# Supplementary material for: Chemokine signaling synchronizes angioblast proliferation and differentiation during pharyngeal arch artery vasculogenesis
Source: Development. 2022 Dec 5;149(23):dev200754. doi: 10.1242/dev.200754 (PMC10114070; doi:10.1242/dev.200754)
Supplement: Supplementary file 1 [file develop-149-200754_review_history.pdf]

Chemokine signaling synchronizes angioblast proliferation and differentiation during pharyngeal arch artery vasculogenesis

Jie Liu, Mingming Zhang, Haojian Dong, Jingwen Liu, Aihua Mao, Guozhu Ning, Yu Cao, Yiyue ZHANG and Qiang Wang
DOI: 10.1242/dev.200754

Editor: Steve Wilson

Review timeline

Original submission:	16 March 2022
Editorial decision:	3 May 2022
First revision received:	7 October 2022
Editorial decision:	1 November 2022
Second revision received:	10 November 2022
Accepted:	14 November 2022

Original submission

First decision letter

MS ID#: DEVELOP/2022/200754

MS TITLE: Chemokine signaling synchronizes angioblast proliferation and differentiation during pharyngeal arch artery vasculogenesis

AUTHORS: Jie Liu, Mingming Zhang, Jingwen Liu, Aihua Mao, Guozhu Ning, Yu Cao, Yiyue ZHANG, and Qiang Wang

I have now received all the referees' reports on the above manuscript, and have reached a decision. The referees' comments are appended below, or you can access them online: please go to BenchPress and click on the 'Manuscripts with Decisions' queue in the Author Area.

As you will see, the referees vary in their opinions on the suitability of the manuscript for publication in Development with reviewer one not supportive. However as the other two reviewers are more enthusiastic, I am happy to give you the opportunity to address the criticisms and concerns of all three reviewers. For instance, it is important to address Reviewer 1's concern that the phenotypes you describe may be secondary consequences of other earlier phenotypes. Please also note that Development will normally permit only one round of major revision.Please attend to all of the reviewers' comments and ensure that you clearly highlight all changes made in the revised manuscript. Please avoid using 'Tracked changes' in Word files as these are lost in PDF conversion. I should be grateful if you would also provide a point-by-point response detailing how you have dealt with the points raised by the reviewers in the 'Response to Reviewers' box. If you do not agree with any of their criticisms or suggestions please explain clearly why this is so.

Reviewer 1

Advance summary and potential significance to field

Liu et al investigate the roles of chemokine signaling in pharyngeal vasculogenesis in zebrafish embryos. Cxcr4a is expressed in pharyngeal angioblasts while its ligand, Cxcl12b, is expressed in

© 2022. Published by The Company of Biologists under the terms of the Creative Commons Attribution License (https://creativecommons.org/licenses/by/4.0/).

neighboring pharyngeal endoderm. The authors show that mutants in Cxcr4a, as well as Cxcl12b, have defects in the formation of posterior pharyngeal arch arteries, which are phenocopied by pharmacological inhibition of downstream effectors such as AKT. Defects include reduced blood flow, angioblast proliferation and differentiation, which the authors argue reflects a novel role for chemokine signaling in coordinating these processes. They also perform biochemical assays in HEK cells to demonstrate that AKT phosphorylates transcription factors required for angioblast commitment and protects them from degradation.

Comments for the author

The biochemical assays provide strong evidence for a direct role of AKT in regulation of Etv2 and Scl protein stability. However, the genetic analyses in zebrafish are less convincing, lack novelty and are not well integrated with the rest of the paper.

Major Comments:
1) Cxcl12b/Cxcr4a signaling is a well-known regulator of vasculogenesis in several contexts, including formation of the lateral dorsal aorta (LDA) in zebrafish embryos. Therefore, it is not surprising that it may also regulate pharyngeal artery formation.

2) Because the posterior pharyngeal arch arteries (PAAs 5 and 6) form later than the LDA, depend on it for blood flow, and are the last of the PAAs to develop, it is extremely difficult to separate primary effects on PAAs from secondary effects.

PAA defects may be secondary to loss of the LDA or simply due to developmental delay. Most of the experiments applying pharmacological inhibitors from 18 hpf onwards, or rescuing phenotypes by restoring gene expression globally, lack temporal specificity. The one exception to this is photocleavable morpholino rescue with Etv2 included in Fig. 6, but even here the authors would need to show that this rescues PAAs without rescuing the LDA. Several other prominent cell types also express Cxcr4b in the pharyngeal arches, including neural crest cells that have been reported to rely on Cxcr4a signaling, which could indirectly influence PAA development and this has not been addressed.

3) The zebrafish studies are not well integrated with the biochemical assays in HEK cells. The paper switches abruptly to in vitro assays testing interactions between AKT, Etv2 and Scl, that are nice, but completely removed from the context of chemokine signaling. PI3K and AKT act downstream of many signals, notably VEGF-mediated angiogenesis. The reduced pAKT staining in Cxcr4b mutants shown in Fig. 4 is suggestive but could also be secondary to loss of other signals or delay.

Minor Comments:

Do all PAA angioblasts express Cxcr4a? In Fig. 1B and C transcripts appear restricted to a small subset of Nkx2.5-expressing angioblasts but this is not discussed.

<u>Reviewer 2</u>

Advance summary and potential significance to field

The publication by Liu et al investigates the influence of cxcr4a chemokine signaling on the formation of the pharyngeal arch arteries (PAAs) in zebrafish embryos. The authors found a specific defect in the 5th and 6th pharyngeal arch in cxcr4a and, to a lesser extent, cxcl12b mutant animals. They continue to show that PI3K signaling was reduced in cxcr4a mutants, affecting the proliferation and differentiation of PAA angioblasts. They back up these findings using several PI3K inhibitors. Through molecular analysis mainly in cultured cells they furthermore show that the PI3K downstream kinase AKT1 affects the stability of both ETV2 and Scl through phosphorylating serine and threonine residues, ultimately leading to protein polyubiquitination and proteasomal degradation. Altogether, the experiments are well documented and quantified and the paper is well written. It elucidates a so far underappreciated aspect of cxcr4a signaling that was previously mainly implicated in guiding endothelial cell migration.

© 2022. Published by The Company of Biologists under the terms of the Creative Commons Attribution License (https://creativecommons.org/licenses/by/4.0/).

Comments for the author

1. The authors claim that PAA endothelial cell migration is unaffected in cxcr4a mutants (Page 8, line 200). However, the authors also show that PAAs 5 and 6 are most strongly affected in cxcr4a mutants. These are the PAAs that migrate, fuse and ultimately connect to the LDA on either side of the embryo (refer to e.g. Nicoli, Lawson et al, Nature 2010, Supplementary Fig. 1C). Is this migration normal in cxcr4a mutant embryos, e.g. do AA5 and AA6 fuse to the LDA in those mutants? If not, migration would be affected. Of note, Nicoli et. al show that the correct migration of PAA cells requires PI3K signaling. Thus, PI3K signaling might also affect migration of PAA cells. The authors need to investigate this possibility, as a failure to connect to the LDA might be the reason that the authors do not observe flow in PAA 5 and PAA6 and not the reduction in angioblast numbers. This needs to be discussed.

2. The authors show pAKT staining in PAA angioblasts (Figure 3A). Almost all angioblasts appear to show pAKT staining. The authors need to validate the antibody specificity, e.g. through blocking AKT phosphorylation using drug treatments and show that this leads to a reduction in pAKT in PAAs (see also below for agonist treatments).

3. The authors show rescue of tie1 expression in cxcr4a mutants after AKT activation using 740-P or SC79 (Figure 3L). Does this treatment lead to an increase in pAKT in PAAs in cxcr4a mutants? Are angioblast numbers also rescued by 740-P or SC79 treatment?

4. The findings that AKT stabilizes ETV2 and SCL are very interesting but are mainly carried out in cultured cells. Antibodies for both proteins are available in zebrafish (Figure 4A, B). The authors need to show that AKT activation/inhibition affects ETV2 and SCL stability in zebrafish embryos. For example, can the authors overexpress FLAG-tagged AKT and activate translation at a later time point using their photo morpholino approach to see whether this stabilizes ETV2 and SCL protein in PAAs? They could also use AKT agonists for this purpose. The tools seem to be available for this.

Reviewer 3

Advance summary and potential significance to field

This manuscript by Liu et al., is well written, interesting, and novel. It contains a comprehensive set of experiment showing that chemokine signalling (cxcl12/cxcr4) in zebrafish activates PI3K/Akt signaling to phosphorylate transcription factors scl/etv2 and prevent these transcription factors from proteosomal degradation, thus allowing the development of pharyngeal arch arteries.

The manuscript first shows that cxcr4a is expressed in the PAAs (yes, and no surprise) and that PAA development is abnormal, particularly in PAAs 5-6, and that proliferation and differentiation of cells is diminished. pAKT appears diminished and the same phenotype can be seen by through PI3K/AKT inhibition. A PI3K agonist and an AKT agonist were able to rescue cxcr4a mutants showing a mechanistic link. They show that AKT promotes stabilization of Etv2 and Scl through kinase activity.

Comments for the author

If cxcl12a/b and cxcr4 are expressed everywhere in vessels, why is the phenotype just observed in the PAAs, particularly the posterior PAAs?

Through the entire manuscript, numbers are lacking from the text or the figure legend. They need to be in one place or other. Currently readers are referred to the graphs and must infer what the numbers are. An example is Figure 2 where the p-values are in the legend, but not the values on the graphs nor the n’s (# experiments and # animals). Please improve the documentation of the experimental results not only in this figure, but all figures.

Line 321: cells are blocked in G1 and yet CDK2 (note this is human nomenclature- please fix) and CDK4/5 are not involved. The authors need a better mechanistic explanation.

© 2022. Published by The Company of Biologists under the terms of the Creative Commons Attribution License (https://creativecommons.org/licenses/by/4.0/).

Figure 1 (line 895); For the images in D, the gata1:DsRed transgenic was used. Was there also tricaine used in this experiment? The wildtype animals look like they have good flow (evidenced by the erythrocytes appearing stacked like coins in the PAAs), but the mutants appear to have stagnant blood in PAA 3/4 . Is this due to a bigger effect of tricaine on heart function in the mutants?

Figure 2: The authors do not comment on the diameter of the PAAs, but this clearly changes over development even in wildtype, and is certainly changed in mutants. Please add this in addition to the cell count data.

Figures 4 is crowded and poorly put together. For instance in figure 4 G-M. This data is presented in a confusing way. What are the numbers above each set of blots? Are these the averages of the blots shown as well as additional blots? This data should be graphed and the blots be either adjacent to the graphs or moved to the supplement. It is very difficult to interpret these blots as they are crowded and the data is not presented in a way that's easy to follow what is happening.

The bands in Figure 5J are not convincing. The authors should replace this image with another replicate. Simialrly in 5H, the second lane shows massive overexpression of the protein (Flag) and it is likely that this is saturated. This is not a good example for quantification. Similarly to Figure 4, graphs might be a better way to present this data (as long as the data is available in the supplement. Schematics might also help a reader understand what the experiment is.

Line 380: the example citing the destruction of beta-catenin after phosphorylation is the opposite of what is happening here where phosphorylation is protective. Please remove this statement, but also please explain why your results are opposite.

Line 436: This sentence does not make sense and is speculative. 'Such spatiotemporal expression of cxcl12b perfectly meets the requirement for activation of Cxcr4a signaling in PAA angioblasts'. You can hypothesize that expression patterns suggest the two can interact, but we have no idea whether this 'perfectly meets the requirement'. This is not scientific. It merely suggests.

Line 977: Figure 4 D and E. In these graphs, Etv2 is destabilize by Akt1 and Scl is stabilized by Akt. Why do the authors conclude that both are stabilized? Is the graph incorrectly labelled?
The biochemical experiments are all done in an overexpression context. Can the authors justify this as opposed to looking at endogenous interactions?

Minor points:
-Figure 1: scale bars are missing in E and F. It appears that the images in F are smaller than those in E, which is why this was noticed. Scale bars should be placed on all images in Figure 1 and following images (for images taken at the same magnification, one scale bar per group of images is fine). The other typical convention is that if the whole animal is shown, we don't usually need a scale bar, but for part of an animal, we'll need to show the scale bar.
-Line 124: potent role- I think you mean potential role.
-Line 203: BrdU cooperation assays should read BrdU incorporation assays.
-Line 293: This sentence does not make sense "Besides, wild-type AKT1, but not its kinase deficient mutant, was also able to promote mouse Etv2 and Scl expression (Fig. 4K)."
- Do you mean that wildtype (no besides needed in this sentence) AKT1 but not its kinase deficient mutant….
-AKT1 is human nomenclature, not fish. Please use the correct species name
-Line 340: should read 'did not enhance'.
-Line 401: is the Scl also FLAG tagged?

© 2022. Published by The Company of Biologists under the terms of the Creative Commons Attribution License (https://creativecommons.org/licenses/by/4.0/).

First revision

Author response to reviewers' comments

Point-to-point responses to reviewers' concerns:

The major changes were highlighted with red color in the revised manuscript.

Reviewer #1

Reviewer 1 Advance Summary and Potential Significance to Field:
Liu et al investigate the roles of chemokine signaling in pharyngeal vasculogenesis in zebrafish embryos. Cxcr4a is expressed in pharyngeal angioblasts while its ligand, Cxcl12b, is expressed in neighboring pharyngeal endoderm. The authors show that mutants in Cxcr4a, as well as Cxcl12b, have defects in the formation of posterior pharyngeal arch arteries, which are phenocopied by pharmacological inhibition of downstream effectors such as AKT. Defects include reduced blood flow, angioblast proliferation and differentiation, which the authors argue reflects a novel role for chemokine signaling in coordinating these processes. They also perform biochemical assays in HEK cells to demonstrate that AKT phosphorylates transcription factors required for angioblast commitment and protects them from degradation.

Reviewer 1 Comments for the Author:

The biochemical assays provide strong evidence for a direct role of AKT in regulation of Etv2 and Scl protein stability. However, the genetic analyses in zebrafish are less convincing, lack novelty and are not well integrated with the rest of the paper.

Response: We thank the reviewer for the professional advices. We have taken many efforts to improve the genetic analyses in zebrafish, such as follows:

1) *cxcl12b*$^{-/-}$ mutants showed obviously reduced p-AKT level in the posterior PAAs (Fig. 9C in the present vision).
2) *cxcr4a* morphants displayed similar decrease of p-AKT expression in the posterior PAAs as observed in *cxcr4a*$^{-/-}$ mutants (Fig. S6C in the present vision).
3) Treatment of wild-type embryos with the AKT-inhibitor, MK-2206, yielded a significant decrease in zEtv2 and zScl expression (Fig. 4C and 4D in the present vision).
4) SC79-mediated reactivation of AKT in *cxcr4a*$^{-/-}$ embryos restored the expression of zEtv2 and zScl proteins (Fig. 4C and 4D in the present vision).

In our revised manuscript, these zebrafish studies have been further integrated with the biochemical assays in HEK293T cells, and provided strong evidences that Cxcr4a is required for the activation of the downstream PI3K/AKT cascade, which facilitates the G1/S cell cycle transition and stabilizes Etv2 and Scl proteins to promote PAA angioblast differentiation. For all the detailed improvements in the current version of our manuscript, please see the highlighted modifications in the text and our responses to all the three reviewers.

Previous reports have demonstrated the function of CXCL12/CXCR4 signaling in vasculogenesis. For example, CXCL12/CXCR4 signaling has been found to play crucial roles in the establishment of organ-specific vascular systems (Ara et al., 2005; Cavallero et al., 2015; Katsumoto and Kume, 2011; Tachibana et al., 1998; Takabatake et al., 2009). In particular, in zebrafish, Cxcl12b/Cxcr4a signaling has been implicated in the formation of the lateral dorsal aorta, arterial-venous connections, and coronary vessels (Bussmann et al., 2011; Harrison et al., 2015; Siekmann et al., 2009). However, most of these previous studies discussed the function of chemokine signaling in guiding endothelial cell migration. Importantly, our study highlights the unique nature of the role of chemokine signaling in governing and coordinating angioblast proliferation and differentiation during PAA morphogenesis. AKT has a well-known role in accelerating cell-cycle progression through activation of Cyclin-dependent kinases. We further reveal that AKT functions downstream of chemokine signaling to phosphorylate and stabilize Etv2 and Scl proteins, thereby promoting PAA

© 2022. Published by The Company of Biologists under the terms of the Creative Commons Attribution License (https://creativecommons.org/licenses/by/4.0/).

angioblast differentiation. Therefore, our study uncovers a so far underappreciated function of Cxcl12b/Cxcr4a in PAA vasculogenesis through orchestrating angioblast proliferation and differentiation.

Major Comments:
1) Cxcl12b/Cxcr4a signaling is a well-known regulator of vasculogenesis in several contexts, including formation of the lateral dorsal aorta (LDA) in zebrafishembryos. Therefore, it is not surprising that it may also regulate pharyngeal artery formation.

Response: As the reviewer mentioned, Cxcl12b/Cxcr4a signaling is a well-known regulator of vasculogenesis. In zebrafish, Cxcl12b/Cxcr4a signaling has been implicated in the formation of the lateral dorsal aorta, arterial-venous connections, and coronary vessels (Bussmann et al., 2011; Harrison et al., 2015; Siekmann et al., 2009). Most of these previous studies discussed the function of chemokine signaling in guiding endothelial cell migration.

However, we found that *cxcr4a* is not required for PAA cell migration. Firstly, we examined PAA angioblast migration using a lineage-tracing analysis in *Tg(nkx2.5:Kaede)* embryos. The Kaede^{+} cells in PAA cluster 5 were specifically photoconverted at 36 hpf. After conversion, their red derivatives were found throughout the PAA5 and sprouted into similar dorsal positions in both the wild-type and mutant embryos at 60 hpf (Fig. S4A and S4B in the present vision). Furthermore, the PAA 5 and PAA 6 of *cxcr4a*$^{-/-}$ mutants fused and ultimately connected to the LDA at 72 hpf (Fig. S4C in the present vision). These observations indicated that deletion of *cxcr4a* did not affect PAA cell migration.

We further found that, genetic ablation of *cxcr4a* severely impaired both the proliferation and differentiation of PAA angioblasts, which ultimately led to PAA stenosis. Follow-up studies revealed that Cxcr4a activated the downstream PI3K/AKT pathway to regulate PAA angioblast growth and differentiation. Biochemical and functional approaches revealed that AKT interacted with and phosphorylated Etv2 and Scl, thereby preventing them from undergoing ubiquitin-mediated proteasomal degradation. Based on these above new findings, we uncovers a novel role of Cxcl12b/Cxcr4a signaling in synchronizing angioblast proliferation and differentiation during pharyngeal arch artery vasculogenesis.

2) Because the posterior pharyngeal arch arteries (PAAs 5 and 6) form later than the LDA, depend on it for blood flow, and are the last of the PAAs to develop, it is extremely difficult to separate primary effects on PAAs from secondary effects. PAA defects may be secondary to loss of the LDA or simply due to developmental delay. Most of the experiments applying pharmacological inhibitors from 18 hpf onwards, or rescuing phenotypes by restoring gene expression globally, lack temporal specificity. The one exception to this is photocleavable morpholino rescue with Etv2 included in Fig. 6, but even here the authors would need to show that this rescues PAAs without rescuing the LDA. Several other prominent cell types also express Cxcr4a in the pharyngeal arches, including neural crest cells that have been reported to rely on Cxcr4a signaling, which could indirectly influence PAA development and this has not been addressed.

Response: Thanks for these constructive suggestions. Exactly, previous studies have shown that *cxcr4a* plays a role in guiding endothelial cell migration during the formation of lateral dorsal aorta (LDA), which carries blood flow from PAAs to the body that is essential for PAA development (Nicoli et al., 2010; Siekmann et al., 2009). Indeed, we found incomplete formation of the LDA in *cxcr4a*$^{-/-}$ mutants at 24 hpf (Fig. S2A in the present vision). However, such defect was gradually restored before or at 48 hpf (Fig. S2A and S2B in the present vision), and the blood flow within LDA appeared normal (Fig. 1D and 1E in the present vision). What's more, in wild-type embryos, PAAs 5 and 6 are lumenized by 50 hpf and exhibit blood flow by 52 hpf (Matthew et al., 2008). Thus, the abnormality of PAAs 5-6 in *cxcr4a*$^{-/-}$ embryos observed at 48 and 60 hpf may not be due to the earlier defects in LDA.

Furthermore, when compared with control animals, *cxcr4a*$^{-/-}$ embryos still showed significantly less blood flow in PAAs 5-6 at 72 hpf (Fig. 1E in the present vision), ruling out the possibility that the PAA defects were resulted from developmental delay.

© 2022. Published by The Company of Biologists under the terms of the Creative Commons Attribution License (https://creativecommons.org/licenses/by/4.0/).

Indeed, most of our pharmacological treatments with signal inhibitors were carried out on embryos from 18 hpf onwards. In the revised manuscript, to further confirm the direct effects of PI3K/AKT signal on the development of posterior PAAs, wild-type embryos were treated with LY294002 or MK-2206 from 36 hpf, when the progenitors in PAA clusters 3-4 have differentiated into angioblasts. As shown in Figure S7C in the present vision, the expression of *tie1* was almost abolished in PAAs 5-6 of inhibitor- treated embryos.

At present, we do have technical difficulties to restore gene expression tissue-specifically in *cxcr4a*$^{-/-}$ embryos. In fact, we used a previously described antisense photo-cleavable morpholino that targeted the N-terminal Flag sequence (AS-Flag- photo-MO) of Flag-zEtv2 and Flag-zScl mRNAs to block their early translation. Embryos were then exposed to UV at 30 hpf to relieve the blocking of mRNA translation. Such temporal ectopic expression experiments demonstrated that the phosphorylation-resistant mutants of zEtv2 and zScl lost their ability to restore *tie1* expression in *cxcr4a*$^{-/-}$ embryos (Fig. 8D-8F in the present vision). Since the defect of LDA formation was gradually restored in *cxcr4a*$^{-/-}$ mutants before or at 48 hpf (Fig. S2A and S2B in the present vision), it is difficult and unnecessary to explore whether temporal ectopic expression of zEtv2 or zScl has rescue effects on LDA formation.

Our recently published studies uncovered an essential role for endodermal pouches in the development of adjacent pharyngeal tissues such as the brachial cartilages and PAAs (Li et al., 2019; Mao et al., 2021; Mao et al., 2019). However, *cxcr4a*$^{-/-}$ mutant embryos exhibited normally developed pouches and craniofacial cartilages (Fig. S1B and S1C in the present vision), indicating that the *cxcr4a* deficient-induced PAA malformation may not be a secondary effect of impaired pouch and head skeleton development. Interestingly, it has been reported that *cxcr4a* is also expressed within the cranial neural crest cells (CNCCs), and *cxcr4a* morphants show aberrant defects in CNCC migration and craniofacial development (Olesnicky Killian et al., 2009). The discrepancy of the role of *cxcr4a* in head cartilage formation between our study and previous report may be due to genetic compensation by other molecules in the *cxcr4a*$^{-/-}$ mutants (Rossi et al., 2015).

3) The zebrafish studies are not well integrated with the biochemical assays in HEK cells. The paper switches abruptly to in vitro assays testing interactions between AKT, Etv2 and Scl that are nice, but completely removed from the context of chemokine signaling. PI3K and AKT act downstream of many signals, notably VEGF-mediated angiogenesis. The reduced pAKT staining in Cxcr4a mutants shown in Fig. 4 is suggestive but could also be secondary to loss of other signals or delay.

Response: Thanks for pointing out this problem. To solve this, we have improved the genetic analyses in zebrafish in the revised manuscript, such as follows: 1) *cxcl12b*$^{-/-}$ mutants showed obviously reduced p-AKT level in the posterior PAAs (Fig. 9C in the present vision). 2) *cxcr4a* morphants displayed similar decrease of p-AKT expression in the posterior PAAs as observed in *cxcr4a*$^{-/-}$ mutants (Fig. S6C in the present vision). 3) Treatment of wild-type embryos with the AKT-inhibitor, MK-2206, yielded a significant decrease in zEtv2 and zScl expression (Fig. 4C and 4D in the present vision). 4) SC79-mediated reactivation of AKT in *cxcr4a*$^{-/-}$ embryos restored the expression of zEtv2 and zScl proteins (Fig. 4C and 4D in the present vision). In our revised manuscript, these zebrafish studies have been further integrated with the biochemical assays in HEK293T cells. Moreover, PI3K/AKT acts downstream of many signals, including Vegfα signaling that guides PAA angiogenesis (Nicoli et al., 2010). However, no significant difference in *vegfa* expression was found between wild-type embryos and *cxcr4a*$^{-/-}$ mutants (Fig. S6D in the present vision). Collectively, these results indicate that the reduced p-AKT staining in *cxcr4a*$^{-/-}$ mutants could not be secondary to loss of other signals or delay, and suggest a role for the PI3K/AKT pathway downstream of Cxcr4a during PAA morphogenesis.

Minor Comments:

Do all PAA angioblasts express Cxcr4a? In Fig. 1B and C transcripts appear restricted to a small subset of Nkx2.5-expressing angioblasts but this is not discussed.

© 2022. Published by The Company of Biologists under the terms of the Creative Commons Attribution License (https://creativecommons.org/licenses/by/4.0/).

Response: Thanks for pointing out this problem. In the "Discussion" section of the present version, we briefly discussed this observation: *cxcl12b* is expressed in the pouch endoderm during PAA development, and its receptor gene *cxcr4a* is expressed in neighboring developing aortic arches. Interestingly, it seems that not all PAA angioblasts express *cxcr4a*, which may be because these cells are in different cell cycle phases or different differentiation stages. Nonetheless, inactivation of *cxcl12b* leads to PAA defects similar to those observed in *cxcr4a*$^{-/-}$ mutants. These observations indicate a conceivable requirement for chemokine ligands from pharyngeal pouches in signal activation and PAA morphogenesis.

Reviewer #2

Reviewer 2 Advance Summary and Potential Significance to Field:
The publication by Liu et al investigates the influence of cxcr4a chemokine signaling on the formation of the pharyngeal arch arteries (PAAs) in zebrafish embryos. The authors found a specific defect in the 5th and 6th pharyngeal arch in cxcr4a and, to a lesser extent, cxcl12b mutant animals. They continue to show that PI3K signaling was reduced in cxcr4a mutants, affecting the proliferation and differentiation of PAA angioblasts. They back up these findings using several PI3K inhibitors. Through molecular analysis mainly in cultured cells they furthermore show that the PI3K downstream kinase AKT1 affects the stability of both ETV2 and Scl through phosphorylating serine and threonine residues, ultimately leading to protein polyubiquitination and proteasomal degradation. Altogether, the experiments are well documented and quantified and the paper is well written. It elucidates a so far underappreciated aspect of cxcr4a signaling that was previously mainly implicated in guiding endothelial cell migration.

Reviewer 2 Comments for the Author:

1. The authors claim that PAA endothelial cell migration is unaffected in cxcr4a mutants (Page 8, line 200). However, the authors also show that PAA 5 and 6 are most strongly affected in cxcr4a mutants. These are the PAAs that migrate, fuse and ultimately connect to the LDA on either side of the embryo (refer to e.g. Nicoli, Lawson et al, Nature 2010, Supplementary Fig. 1C). Is this migration normal in cxcr4a mutant embryos, e.g. do AA5 and AA6 fuse to the LDA in those mutants? If not, migration would be affected. Of note, Nicoli et. al show that the correct migration of PAA cells requires PI3K signaling. Thus, PI3K signaling might also affect migration of PAA cells. The authors need to investigate this possibility, as a failure to connect to the LDA might be the reason that the authors do not observe flow in PAA 5 and PAA 6 and not the reduction in angioblast numbers. This needs to be discussed.

Response: This is a good point. To explore whether PAA cell migration is affected in *cxcr4a*$^{-/-}$ mutants, we firstly examined PAA angioblast migration using a lineage-tracing analysis in *Tg(nkx2.5:Kaede)* embryos. The Kaede^{+} cells in PAA cluster 5 were specifically photoconverted at 36 hpf. After conversion, their red derivatives were found throughout the PAA5 and sprouted into similar dorsal positions in both the wild-type and mutant embryos at 60 hpf (Fig. S4A and S4B in the present vision). In the revised manuscript, we further found that the PAA 5 and PAA 6 of *cxcr4a*$^{-/-}$ mutants fused and ultimately connected to the LDA at 72 hpf (Fig. S4C in the present vision). Moreover, when compared with control animals, *cxcr4a*$^{-/-}$ embryos showed significantly less blood flow in PAAs 5 and 6 at 72 hpf (Fig. 1E in the present vision), further supporting the connection of PAAs 5 and 6 and the LDA. These results suggest that *cxcr4a* is not required for angioblast migration.

It has been reported that blood flow-triggered PI3K/AKT signaling is required for the correct migration of PAA cells (Nicoli et al., 2010). However, PAA angioblasts in *cxcr4a*$^{-/-}$ mutants display no migration defects. PAAs 5 and 6 are lumenized by 50 hpf and exhibit blood flow by 52 hpf (Matthew et al., 2008; Nicoli et al., 2010). In our study, the p-AKT expression is profoundly reduced in the PAAs of *cxcr4a*$^{-/-}$ mutants before or at 48 hpf. Thus, PI3K/AKT pathway may be activated by diversified upstream signals at different developmental stages and perform distinct functions during PAA morphogenesis. For details, please to see the "Discussion" section.

© 2022. Published by The Company of Biologists under the terms of the Creative Commons Attribution License (https://creativecommons.org/licenses/by/4.0/).

2. The authors show pAKT staining in PAA angioblasts (Figure 3A). Almost all angioblasts appear to show pAKT staining. The authors need to validate the antibody specificity, e.g. through blocking AKT phosphorylation using drug treatments and show that this leads to a reduction in pAKT in PAAs (see also below for agonist treatments).
Response: Thanks for this suggestion. We used AKT inhibitor, MK-2206, to inhibit the AKT phosphorylation in wild-type embryos to validate the antibody specificity. As shown in Fig. S6B in the present vision, p-AKT expression was found in all the PAA angioblasts of control animals, but profoundly repressed in the PAAs 3-6 of embryos treated with 10 μM MK-2206, suggesting the fine specificity of the antibody we used in our study.

3. The authors show rescue of tie1 expression in cxcr4a mutants after AKT activation using 740-P or SC79 (Figure 3L). Does this treatment lead to an increase in pAKT in PAAs in cxcr4a mutants? Are angioblast numbers also rescued by 740-P or SC79 treatment?
Response: This is a good question. In the revised manuscript, use of the AKT agonist SC79 clearly restored the phosphorylation level of AKT and the numbers of PAA cells in *cxcr4a*$^{-/-}$ mutants (Fig. S7D; Fig. 3J and 3K in the present vision).

4. The findings that AKT stabilizes ETV2 and SCL are very interesting but are mainly carried out in cultured cells. Antibodies for both proteins are available in zebrafish (Figure 4A, B). The authors need to show that AKT activation/inhibition affects ETV2 and SCL stability in zebrafish embryos. For example, can the authors overexpress FLAG-tagged AKT and activate translation at a later time point using their photo morpholino approach to see whether this stabilizes ETV2 and SCL protein in PAAs? They could also use AKT agonists for this purpose. The tools seem to be available for this.
Response: Thanks for these constructive suggestions. According to the reviewer's suggestion, wild-type embryos were treated with AKT inhibitor MK-2206 and *cxcr4a*$^{-/-}$ mutants were treated with AKT agonist SC79, and the zEtv2 and zScl protein levels were examined by immunofluorescence staining. Treatment of wild-type embryos with the AKT-inhibitor, MK-2206, yielded a significant decrease in zEtv2 and zScl expression (Fig. 4C and 4D in the present vision). Notably, SC79-mediated reactivation of AKT in *cxcr4a*$^{-/-}$ embryos restored the expression of these proteins (Fig. 4C and 4D in the present vision). Overall, these observations indicate that chemokine signaling has a function in regulating the protein stability of both zEtv2 and zScl through PI3K/AKT pathway.

Reviewer #3

Reviewer 3 Advance Summary and Potential Significance to Field:

This manuscript by Liu et al., is well written, interesting, and novel. It contains a comprehensive set of experiment showing that chemokine signaling (cxcl12/cxcr4) in zebrafish activates PI3K/Akt signaling to phosphorylate transcription factors scl/etv2 and prevent these transcription factors from proteosomal degradation, thus allowing the development of pharyngeal arch arteries. The manuscript first shows that cxcr4a is expressed in the PAAs (yes, and no surprise) and that PAA development is abnormal, particularly in PAAs 5 and 6, and that proliferation and differentiation of cells is diminished. pAKT appears diminished and the same phenotype can be seen by through PI3K/AKT inhibition. A PI3K agonist and an AKT agonist were able to rescue cxcr4a mutants showing a mechanistic link. They show that AKT promotes stabilization of Etv2 and Scl through kinase activity.

Reviewer 3 Comments for the Author:

If cxcl12a/b and cxcr4 are expressed everywhere in vessels, why is the phenotype just observed in the PAAs, particularly the posterior PAAs?
Response: Several previous reports have demonstrated the function of CXCL12/CXCR4 signaling in the establishment of organ-specific vascular systems (Ara et al., 2005; Cavallero et al., 2015; Katsumoto and Kume, 2011; Tachibana et al., 1998; Takabatake et al., 2009). In particular, in zebrafish, Cxcl12b/Cxcr4a signaling has been implicated in the formation of the lateral dorsal aorta, arterial-venous connections, and coronary vessels (Bussmann et al., 2011; Harrison et al., 2015; Siekmann et al., 2009). Thus, *cxcr4a*$^{-/-}$ mutants display vascular defects not only in the PAAs,

© 2022. Published by The Company of Biologists under the terms of the Creative Commons Attribution License (https://creativecommons.org/licenses/by/4.0/).

but also in the lateral dorsal aort, posterior cerebrovascular, and coronary vasculature. However, most of previous studies discussed the function of chemokine signaling in guiding endothelial cell migration. Importantly, our study highlights the unique nature of the role of chemokine signaling in governing and coordinating angioblast proliferation and differentiation during PAA morphogenesis.

Genetic ablation of *cxcr4a* only affected the angioblast differentiation in PAAs 5 and 6 in most embryos, implying different sensitivities to the inactivation of Cxcr4a signaling between anterior and posterior PAAs. Interestingly, wild-type embryos treated with lower concentrations of either PI3K or AKT inhibitors led to a phenotype similar to *cxcr4a*$^{-/-}$ mutants. However, treatment with higher inhibitor concentrations induced significant defects in angioblast differentiation in all PAAs. These findings indicate a possibility that in addition to Cxcr4a signaling, there remain other unidentified signaling mechanisms that activate the PI3K/AKT cascade and facilitate PAA formation.

Through the entire manuscript, numbers are lacking from the text or the figure legend. They need to be in one place or other. Currently readers are referred to the graphs and must infer what the numbers are. An example is Figure 2 where the p-values are in the legend, but not the values on the graphs nor the n's (# experiments and # animals). Please improve the documentation of the experimental results not only in this figure, but all figures.

Response: Thanks for this suggestion. In the revised manuscript, in order to make it easier for readers to read the Figures and understand relevant information, we have put all the numbers of *p*-values and animals on the relevant graphs.

Line 321: cells are blocked in G1 and yet CDK2 (note this is human nomenclature- please fix) and CDK4/6 are not involved. The authors need a better mechanistic explanation.
Response: We are sorry for the inaccurate statement. In the revised manuscript, we have modified the related description as follows: To our surprise and despite an apparent reduction in PAA cell numbers in the resulting embryos (Fig. S9C and S9D), the angioblast differentiation of PAA cells remained unaffected as revealed by *tie1* expression (Fig. S9E). These observations demonstrate that both CDK2 and CDK4/6 are necessary for PAA cell proliferation, but not required for angioblast differentiation. These results also open the possibility that AKT might directly phosphorylate Etv2 and Scl to repress their turnover. (Because the function of CDKs in the cell cycles of zebrafish, mouse, and human cells are very conservative, CDK2 and CDK4/6 are generally used in most parts of the manuscript.)

Figure 1 (line 895); For the images in D, the gata1:DsRed transgenic was used. Was there also tricaine used in this experiment? The wildtype animals look like they have good flow (evidenced by the erythrocytes appearing stacked like coins in the PAAs), but the mutants appear to have stagnant blood in PAA 3/4. Is this due to a bigger effect of tricaine on heart function in the mutants?

Response: This is a good question. Exactly, wild-type and *cxcr4a*$^{-/-}$ mutant embryos bearing *Tg(nkx2.5:ZsYellow;gata1:DsRed)* transgene used in this experiment were treated with tricaine according to usual practice. But the heart beating and the blood flow were not obviously disturbed by tricaine treatment. It seemed that PAAs 3 and 4 of *cxcr4a*$^{-/-}$ mutants at 60 hpf carried a slightly less blood flow (Fig. 1D in the present vision). However, at 72 hpf, when compared with control animals, *cxcr4a*$^{-/-}$ embryos showed much normal blood flow in PAAs 3 and 4 and still significantly less blood flow in PAAs 5 and 6 (Fig. 1E in the present vision). These results demonstrate a critical role of *cxcr4a* in the development of posterior PAAs, and rule out side effect of tricaine treatment on heart function in the mutants.

Figure 2: The authors do not comment on the diameter of the PAAs, but this clearly changes over development even in wildtype, and is certainly changed in mutants. Please add this in addition to the cell count data.
Response: Thanks for this suggestion. Because the PAAs 5 and 6 have not yet begun to lumen at 48 hpf, we only measured the diameters of the PAAs at 60 hpf. As shown in Fig. S3 in the present vision, loss of *cxcr4a* obviously reduced the diameters of PAAs 5 and 6.

© 2022. Published by The Company of Biologists under the terms of the Creative Commons Attribution License (https://creativecommons.org/licenses/by/4.0/).

Figures 4 is crowded and poorly put together. For instance in figure 4 G-M. This data is presented in a confusing way. What are the numbers above each set of blots? Are these the averages of the blots shown as well as additional blots? This data should be graphed and the blots be either adjacent to the graphs or moved to the supplement. It is very difficult to interpret these blots as they are crowded and the data is not presented in a way that's easy to follow what is happening.
Response: We are sorry for not showing these results accurately. We have changed the layout of these Figures. As shown in Fig. 5 and Fig. 7 in the present vision, the results of blots have been graphed as column charts. Moreover, the column charts have been shown adjacent to the related blots. In this way, the reader can easily understand these results.

The bands in Figure 5J are not convincing. The authors should replace this image with another replicate. Simialrly in 5H, the second lane shows massive overexpression of the protein (Flag) and it is likely that this is saturated. This is not a good example for quantification. Similarly to Figure 4, graphs might be a better way to present this data (as long as the data is available in the supplement. Schematics might also help a reader understand what the experiment is.
Response: Thanks for these suggestions. Fig. 5H and Fig. 5J have been replace with more suitable replicates in the revised manuscript. For details, please to see Fig. 7E and 7H in the present version.

Line 380: the example citing the destruction of beta-catenin after phosphorylation is the opposite of what is happening here where phosphorylation is protective. Please remove this statement, but also please explain why your results are opposite.
Response: Thanks for pointing out this problem. Protein post-translational modifications (PTMs) can alter protein properties, such as structure, location, turnover, and so on, leading to diverse functions. As two important PTMs, the interplay between ubiquitination and phosphorylation has emerged as a prominent posttranslational crosstalk. A recurring theme between them is that phosphorylation often influences the ubiquitination and thus degradation of the protein (Cell Communication and Signaling. 2013; 11:52). More phosphorylation-promoted degradation were discovered over the years, but the situation was not absolute. For example, the proto-oncogene c-Jun is protected from ubiquitin-dependent degradation after phosphorylation by MAP kinases, and Phosphorylation of Pah1 inhibited its degradation (Journal of Biological Chemistry, 2015; Science, 1997). Moreover, it has been reported phosphorylation delays turnover for many proteins in growing cells (Developmental Cell, 2021). Therefore, phosphorylation modification of different proteins can either enhance or inhibit their degradation.

It is well known that when Wnt signal is absent, β-Catenin will be phosphorylated by glycogen synthase kinase-3β, and then destined for ubiquitin-mediated degradation (Liu et al., 2002). In our study, we merely used as a positive control that the degradation of β-Catenin could be inhibited by overexpressing Ub K48R/G76A, a dominant negative inhibitor of poly-Ub chain formation, to examine whether the degradation of Scl is related to ubiquitination. In order to avoid unnecessary misunderstanding, we have revised the original description as follows: By overexpressing Ub K48R/G76A, a dominant negative inhibitor of poly-Ub chain formation, we found that the decay of zEtv2 and zScl proteins was distinctly repressed with an efficiency comparable to that of β-Catenin (Fig. 8A), which is destined for ubiquitin-mediated degradation when Wnt signal is absent. For details, please to see the text of the present vision.

Line 436: This sentence does not make sense and is speculative. 'Such spatiotemporal expression of cxcl12b perfectly meets the requirement for activation of Cxcr4a signaling in PAA angioblasts'. You can hypothesize that expression patterns suggest the two can interact, but we have no idea whether this 'perfectly meets the requirement'. This is not scientific. It merely suggests.
Response: We agree with this point. This sentence has been modified as the follow: These results imply that pouch endoderm-expressed *cxcl12b* might be responsible for activation of Cxcr4a signaling in PAA angioblasts.

Line 977: Figure 4 D and E. In these graphs, Etv2 is destabilize by Akt1 and Scl is stabilized by Akt. Why do the authors conclude that both are stabilized? Is the graph incorrectly labelled?
Response: Thanks for pointing out this problem. Actually, both Etv2 and Scl are stabilized by AKT1. The graph of Fig. 4D in the previous version was incorrectly labelled, and we are sorry for this mistake. In the revised manuscript, this error has been corrected in Fig. 4F in the present version.

The biochemical experiments are all done in an overexpression context. Can the authors justify this as opposed to looking at endogenous interactions?

© 2022. Published by The Company of Biologists under the terms of the Creative Commons Attribution License (https://creativecommons.org/licenses/by/4.0/).

Response: This is a good point. Because the antibody against endogenous zebrafish Akt1 is currently unavailable, we injected *Flag-AKT1* mRNA into wild-type embryos, and the resulting embryos were harvested at 38 hpf for co-immunoprecipitation assays. As revealed in Fig. 6C in the present version, overexpressed Flag-AKT1 could interact with endogenous zEtv2 and zScl.

Minor points:

-Figure 1: scale bars are missing in E and F. It appears that the images in F are smaller than those in E, which is why this was noticed. Scale bars should be placed on all images in Figure 1 and following images (for images taken at the same magnification, one scale bar per group of images is fine). The other typical convention is that if the whole animal is shown, we don't usually need a scale bar, but for part of an animal, we'll need to show the scale bar.
Response: Thanks for this suggestion. The scale bars have been provided where they are needed in all the images.

-Line 124: potent role- I think you mean potential role.
-Line 203: BrdU cooperation assays should read BrdU incorporation assays. **Response:** Thanks for pointing out these problems. We have corrected these two mistakes in the related sentences.

-Line 293: This sentence does not make sense "Besides, wild-type AKT1, but not its kinase deficient mutant, was also able to promote mouse Etv2 and Scl expression (Fig. 4K)." Do you mean that wildtype (no besides needed in this sentence) AKT1 but not its kinase deficient mutant….
Response: Thanks for this suggestion. We have modified the sentence as follow: Furthermore, wild-type AKT1—but not its kinase deficient mutant—was also able to promote mouse Etv2 and Scl expression.

-AKT1 is human nomenclature, not fish. Please use the correct species name.
Response: We thank the reviewer for pointing this out. AKT proteins are highly conserved in vertebrates, including zebrafish, mouse, and human. For this reason, human AKT1 was used in our related experiments.

-Line 340: should read 'did not enhance'.
Response: We are sorry for this grammatical mistake. The sentence has been corrected in the present version.

-Line 401: is the Scl also FLAG tagged?
Response: Thanks for pointing out the mistake.In fact, the Scl was also FLAG tagged. We have changed "*Flag-zEtv2* and *zScl* mRNAs" to "*Flag-zEtv2* and *Flag-zScl* mRNAs".

Second decision letter

MS ID#: DEVELOP/2022/200754

MS TITLE: Chemokine signaling synchronizes angioblast proliferation and differentiation during pharyngeal arch artery vasculogenesis

AUTHORS: Jie Liu, Mingming Zhang, Haojian Dong, Jingwen Liu, Aihua Mao, Guozhu Ning, Yu Cao, Yiyue ZHANG, and Qiang Wang

I have now received all the referees reports on the above manuscript, and you will be pleased to see that the referees are mostly happy with your revisions and that there are just a few minor issues to consider before publication. The referees' comments are appended below, or you can access them online: please go to BenchPress and click on the 'Manuscripts with Decisions' queue in the Author Area.

© 2022. Published by The Company of Biologists under the terms of the Creative Commons Attribution License (https://creativecommons.org/licenses/by/4.0/).

Reviewer 1

Advance summary and potential significance to field
Liu et al investigate the roles of chemokine signaling in pharyngeal vasculogenesis in zebrafish embryos. Cxcr4a is expressed in pharyngeal angioblasts while its ligand, Cxcl12b, is expressed in neighboring pharyngeal endoderm. The authors show that mutants in Cxcr4a, as well as Cxcl12b, have defects in the formation of posterior pharyngeal arch arteries, which are phenocopied by pharmacological inhibition of downstream effectors such as AKT. Defects include reduced blood flow, angioblast proliferation and differentiation, which the authors argue reflects a novel role for chemokine signaling in coordinating these processes. They also perform biochemical assays in HEK cells to demonstrate that AKT phosphorylates transcription factors required for angioblast commitment and protects them from degradation.

Comments for the author

The authors have addressed most of my major concerns and the paper is greatly improved. The additional data in zebrafish show convincingly that the pharyngeal vascular phenotypes are not simply due to migration defects or developmental delay. They are also now better integrated with the in vitro biochemical assays.

Reviewer 2

Advance summary and potential significance to field

No change from my initial assessment.

Comments for the author

The authors have addressed most of my concerns and greatly improved the paper. However, I asked whether angioblast numbers were increased after 740 Y-P or SC-79 treatment (point 3). In the revised version, the authors now only provide data for SC-79 treatment. Is there a reason, why the authors do not provide data on 740 Y-P? I also did not find information on 740 Y-P treatment in the methods section (Pharmacological treatments), even though the authors show 740 Y-P treatment in Figure 3K. A quick check of the provided reference (Hsieh, 2018) seems to refer to a publication that did not use 740 Y-P? The authors need to clarify these points.

The authors might want to cite the publication by Stückemann et al. (Aanstad), Development 139:2711-20, 2012 showing that cxcr4a influences proliferation of endodermal cells as another example where cxcr4a signaling does not influence cell migration but proliferation.

Reviewer 3

Advance summary and potential significance to field

This manuscript by Liu et al., is well written, interesting, and novel. It contains a comprehensive set of experiment showing that chemokine signalling (cxcl12/cxcr4) in zebrafish activates PI3K/Akt signaling to phosphorylate transcription factors Scl and Etv2 and prevent these transcription factors from proteosomal degradation, thus allowing the development of pharyngeal arch arteries.

The manuscript first shows that cxcr4a is expressed in the PAAs (yes, and no surprise) and that PAA development is abnormal, particularly in PAAs 5 and 6, and that proliferation and differentiation of cells is diminished. pAKT appears diminished and the same phenotype can be seen by through PI3K/AKT inhibition. A PI3K agonist and an AKT agonist were able to rescue cxcr4a mutants showing a mechanistic link. They show that AKT promotes stabilization of Etv2 and Scl through kinase activity.

© 2022. Published by The Company of Biologists under the terms of the Creative Commons Attribution License (https://creativecommons.org/licenses/by/4.0/).

Comments for the author

The authors have attentively addressed my concerns.

Second revision

Author response to reviewers' comments

Point-to-point responses to reviewers' concerns:

The major changes were highlighted with red color in the revised manuscript.

Reviewer 1

Reviewer 1 Advance Summary and Potential Significance to Field:

Liu et al investigate the roles of chemokine signaling in pharyngeal vasculogenesis in zebrafish embryos. Cxcr4a is expressed in pharyngeal angioblasts while its ligand, Cxcl12b, is expressed in neighboring pharyngeal endoderm. The authors show that mutants in Cxcr4a, as well as Cxcl12b, have defects in the formation of posterior pharyngeal arch arteries, which are phenocopied by pharmacological inhibition of downstream effectors such as AKT. Defects include reduced blood flow, angioblast proliferation and differentiation, which the authors argue reflects a novel role for chemokine signaling in coordinating these processes. They also perform biochemical assays in HEK cells to demonstrate that AKT phosphorylates transcription factors required for angioblast commitment and protects them from degradation.

Reviewer 1 Comments for the Author:

The authors have addressed most of my major concerns and the paper is greatly improved. The additional data in zebrafish show convincingly that the pharyngeal vascular phenotypes are not simply due to migration defects or developmental delay. They are also now better integrated with the in vitro biochemical assays.
Response: We thank you very much for your gracious comment.

Reviewer 2

Reviewer 2 Advance Summary and Potential Significance to Field:
No change from my initial assessment.

Reviewer 2 Comments for the Author:

The authors have addressed most of my concerns and greatly improved the paper. However, I asked whether angioblast numbers were increased after 740 Y-P or SC-79 treatment (point 3). In the revised version, the authors now only provide data for SC- 79 treatment. Is there a reason, why the authors do not provide data on 740 Y-P? I also did not find information on 740 Y-P treatment in the methods section (Pharmacological treatments), even though the authors show 740 Y-P treatment in Figure 3K. A quick check of the provided reference (Hsieh, 2018) seems to refer to a publication that did not use 740 Y-P? The authors need to clarify these points.
Response: We are very sorry that we mistakenly thought that the data for SC-79 treatment would answer the reviewer's concerns. In the revised manuscript, *cxcr4a*$^{-/-}$ mutants were further treated with or without the PI3K agonist 740Y-P, and the changes in the phosphorylation level of AKT and the numbers of PAA cells were examined. To save space, we combined these results with previous results about SC-79 treatment. As shown in Fig. S7D and Fig. 3J-3N in the present vision, we treated the *cxcr4a*$^{-/-}$ mutants with either 740Y-P or SC79, which are potent agonists of PI3K and AKT, respectively (Hsieh et al., 2018; Wolman et al., 2015). Use of these agonists clearly restored the

© 2022. Published by The Company of Biologists under the terms of the Creative Commons Attribution License (https://creativecommons.org/licenses/by/4.0/).

phosphorylation level of AKT, the numbers of PAA cells, and the formation of PAAs in *cxcr4a*$^{-/-}$ mutants. Taken together, these results suggest that Cxcr4a promotes PAA development through its downstream PI3K/AKT signal cascade.

In addition, the reference (Hsieh et al., 2018) refers to a publication using AKT agonist SC79, while the reference (Wolman et al., 2015) refers to a publication that both the PI3K agonist 740Y-P and AKT agonist SC79 were used.

We are sorry for the lacking of the description of 740 Y-P treatment in the methods section. The information on 740 Y-P treatment has been provided in the methods section as follow: To activate PI3K/AKT activity, embryos were treated with 1 μM 740 Y-P (1236188-16-1, R&D Systems) or 0.5 μM SC79 (SF2730, Beyotime) from 18 hpf until harvest, respectively, and HeLa cells were treated with 10 μM SC79 for 2 hours prior to harvest.

The authors might want to cite the publication by Stückemann et al. (Aanstad), Development 139:2711-20, 2012 showing that cxcr4a influences proliferation of endodermal cells as another example where cxcr4a signaling does not influence cell migration but proliferation.
Response: Thanks for this suggestion. This publication has been cited in the revised manuscript: It has been shown that *cxcr4a* plays a major role in promoting endodermal cell proliferation (Stuckemann et al., 2012). Therefore, we then performed bromodeoxyuridine (BrdU) incorporation assays to examine whether *cxcr4a* also regulates the proliferation of PAA angioblasts. Coincidentally, we found a considerable decline in the proliferating ability of *cxcr4a*-depleted cells within PAAs 5 and 6 (Fig. 2E and 2F in the present vision).

Reviewer 3

Reviewer 3 Advance Summary and Potential Significance to Field:
This manuscript by Liu et al., is well written, interesting, and novel. It contains a comprehensive set of experiment showing that chemokine signalling (cxcl12/cxcr4) in zebrafish activates PI3K/Akt signaling to phosphorylate transcription factors scl/etv2 and prevent these transcription factors from proteosomal degradation, thus allowing the development of pharyngeal arch arteries.

The manuscript first shows that cxcr4a is expressed in the PAAs (yes, and no surprise) and that PAA development is abnormal, particularly in PAAs 5 and 6, and that proliferation and differentiation of cells is diminished. pAKT appears diminished and the same phenotype can be seen by through PI3K/AKT inhibition. A PI3K agonist and an AKT agonist were able to rescue cxcr4a mutants showing a mechanistic link. They show that AKT promotes stabilization of Etv2 and Scl through kinase activity.

Reviewer 3 Comments for the Author:

The authors have attentively addressed my concerns.
Response: We thank you very much for your gracious comment.

Third decision letter

MS ID#: DEVELOP/2022/200754

MS TITLE: Chemokine signaling synchronizes angioblast proliferation and differentiation during pharyngeal arch artery vasculogenesis

AUTHORS: Jie Liu, Mingming Zhang, Haojian Dong, Jingwen Liu, Aihua Mao, Guozhu Ning, Yu Cao, Yiyue ZHANG, and Qiang Wang
ARTICLE TYPE: Research Article

I am happy to tell you that your manuscript has been accepted for publication in Development, pending our standard ethics checks.

© 2022. Published by The Company of Biologists under the terms of the Creative Commons Attribution License (https://creativecommons.org/licenses/by/4.0/).
